# The COMMD3/8 complex determines GRK6 specificity for chemoattractant receptors

**DOI:** 10.1084/jem.20181494

**Published:** 2019-05-14

**Authors:** Akiko Nakai, Jun Fujimoto, Haruhiko Miyata, Ralf Stumm, Masashi Narazaki, Stefan Schulz, Yoshihiro Baba, Atsushi Kumanogoh, Kazuhiro Suzuki

**Affiliations:** 1 Laboratory of Immune Response Dynamics, Immunology Frontier Research Center, Osaka University, Osaka, Japan; 2 Department of Respiratory Medicine and Clinical Immunology, Graduate School of Medicine, Osaka University, Osaka, Japan; 3 Department of Experimental Genome Research, Research Institute for Microbial Diseases, Osaka University, Osaka, Japan; 4 Institute of Pharmacology and Toxicology, Jena University Hospital, Friedrich Schiller University Jena, Jena, Germany; 5 Division of Immunology and Genome Biology, Medical Institute of Bioregulation, Kyushu University, Fukuoka, Japan; 6 Department of Molecular Immunology, Research Institute for Microbial Diseases, Osaka University, Osaka, Japan

## Abstract

Lymphocyte migration is mediated by G protein–coupled receptors (GPCRs) that respond to chemoattractive molecules. After their activation, GPCRs are phosphorylated by different GPCR kinases (GRKs), which produces distinct functional outcomes through β-arrestins. However, the molecular machinery that targets individual GRKs to activated GPCRs remains elusive. Here, we identified a protein complex consisting of copper metabolism MURR1 domain–containing (COMMD) 3 and COMMD8 (COMMD3/8 complex) as an adaptor that selectively recruits a specific GRK to chemoattractant receptors and promotes lymphocyte chemotaxis. COMMD8, whose stability depended on COMMD3, was recruited to multiple chemoattractant receptors. Deficiency of COMMD8 or COMMD3 impaired B cell migration and humoral immune responses. Using CXC-chemokine receptor 4 (CXCR4) as a model, we demonstrated that the COMMD3/8 complex selectively recruited GRK6 and induced GRK6-mediated phosphorylation of the receptor and activation of β-arrestin–mediated signaling. Thus, the COMMD3/8 complex is a specificity determinant of GRK targeting to GPCRs and represents a point of regulation for immune responses.

## Abstract (precis)

Nakai et al. show that the COMMD3/8 complex functions as an adaptor that selectively recruits GRK6 to chemoattractant receptors and promotes B cell migration and humoral immune responses.

## Graphical Abstract

**Figure:**
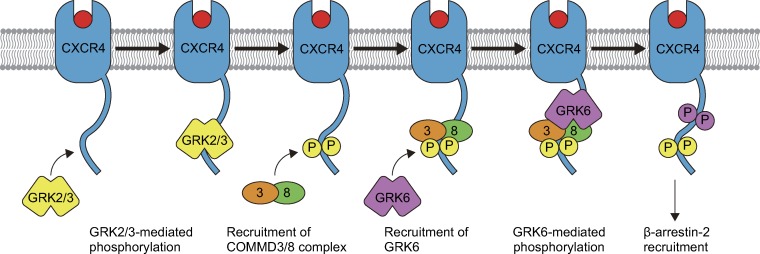

## Introduction

G protein–coupled receptors (GPCRs) comprise the largest family of surface receptors and regulate a broad spectrum of biological processes (Lefkowitz, 2007). GPCRs for chemoattractants, represented by chemokine receptors, play essential roles in the immune system by orchestrating cell trafficking among various anatomical sites (Rot and von Andrian, 2004; Griffith et al., 2014). Blood-circulating lymphocytes enter secondary lymphoid organs, including the spleen, LNs, and Peyer’s patches, and migrate to separate subcompartments where they survey for antigens. After spending several hours to a day in a secondary lymphoid organ, lymphocytes exit to travel to other lymphoid organs and continue antigen surveillance. Once lymphocytes encounter cognate antigens, they rapidly change their migration program to efficiently induce immune responses in the lymphoid organ. Each of these steps of lymphocyte trafficking is controlled by a specific chemoattractant receptor(s). In the case of B cells, their LN entry is mediated by CC-chemokine receptor 7 (CCR7) and CXC-chemokine receptor 4 (CXCR4; Förster et al., 1999; Okada et al., 2002). CXCR5 is essential for B cell localization in lymphoid follicles (Förster et al., 1996; Ansel et al., 2000), which is a prerequisite for antigen encounter of the cells (Junt et al., 2005; Suzuki et al., 2009). Both B and T cells strongly depend on sphingosine-1-phosphate (S1P) receptor 1 (S1PR1) for their LN egress (Matloubian et al., 2004). Following initial antigen encounter, B cells up-regulate the expression of the oxysterol receptor Epstein-Barr virus–induced gene 2 (EBI2; also known as GPR183; Gatto et al., 2009; Pereira et al., 2009) and CCR7 (Reif et al., 2002; Okada and Cyster, 2006), which directs their positioning to facilitate further acquisition of the antigen and encounters with cognate T cells and thereby promotes humoral immune responses.

Agonist binding to GPCRs activates heteromeric G proteins to regulate the generation of second messengers that modulate downstream signaling and consequential physiological responses. Agonist-occupied GPCRs are phosphorylated by GPCR kinases (GRKs) and subsequently recruit β-arrestins, which leads to termination of G protein–mediated signaling and internalization of the receptors (Lefkowitz and Shenoy, 2005; Reiter and Lefkowitz, 2006). Receptor-bound β-arrestins also serve as scaffolds to activate signaling molecules, including MAPKs. The GRK family consists of seven mammalian members, among which GRK2, GRK3, GRK5, and GRK6 are expressed ubiquitously (Pitcher et al., 1998; Ferguson, 2001). Different GRKs phosphorylate distinct sites on the receptor C terminus, establishing a barcode that dictates the functional consequences of β-arrestin engagement (Busillo et al., 2010; Butcher et al., 2011; Nobles et al., 2011). Thus, specific targeting of GRKs to activated GPCRs is crucial for signal transduction. However, the molecular basis that determines the specificity of GRK targeting is poorly understood. Here, we show that a protein complex consisting of copper metabolism MURR1 domain–containing (COMMD) 3 and COMMD8 selectively recruits GRK6 to chemoattractant receptors and promotes chemotactic migration of B cells. The COMMD3/8 complex also mediates signaling of the β_2_-adrenergic receptor (β_2_AR) in the same manner, suggesting that this protein complex functions as a specificity determinant of GRK targeting to a wide range of GPCRs.

## Results

### The COMMD3/8 complex interacts with chemoattractant receptors

In search of factors involved in GRK recruitment to chemoattractant receptors, we performed yeast two-hybrid screening of a cDNA library from human bone marrow and identified COMMD8 as a protein that binds to the C-terminal amino acid sequence (residues 306–352) of human CXCR4. Additional screening revealed an interaction of COMMD8 with COMMD3. The COMMD protein family consists of 10 members, which are homologous to MURR1 (COMMD1), the product of a gene responsible for copper toxicosis in a dog breed, and defined by the presence of the conserved COMM domain in their C termini (Burstein et al., 2005). Eight of the COMMD proteins, other than COMMD8 and COMMD3, have been suggested to act as negative regulators of NF-κB (Ganesh et al., 2003; Burstein et al., 2005). In mammals, COMMD8 and COMMD3 are highly conserved, relatively small proteins with respective lengths of 183 and 195 amino acids without any characterized domains besides the COMM domain (Fig. S1 A), and their genes are expressed ubiquitously (BioGPS at http://biogps.org; Burstein et al., 2005). However, the functions of COMMD8 and COMMD3 are largely unclear.

**Figure 1.**
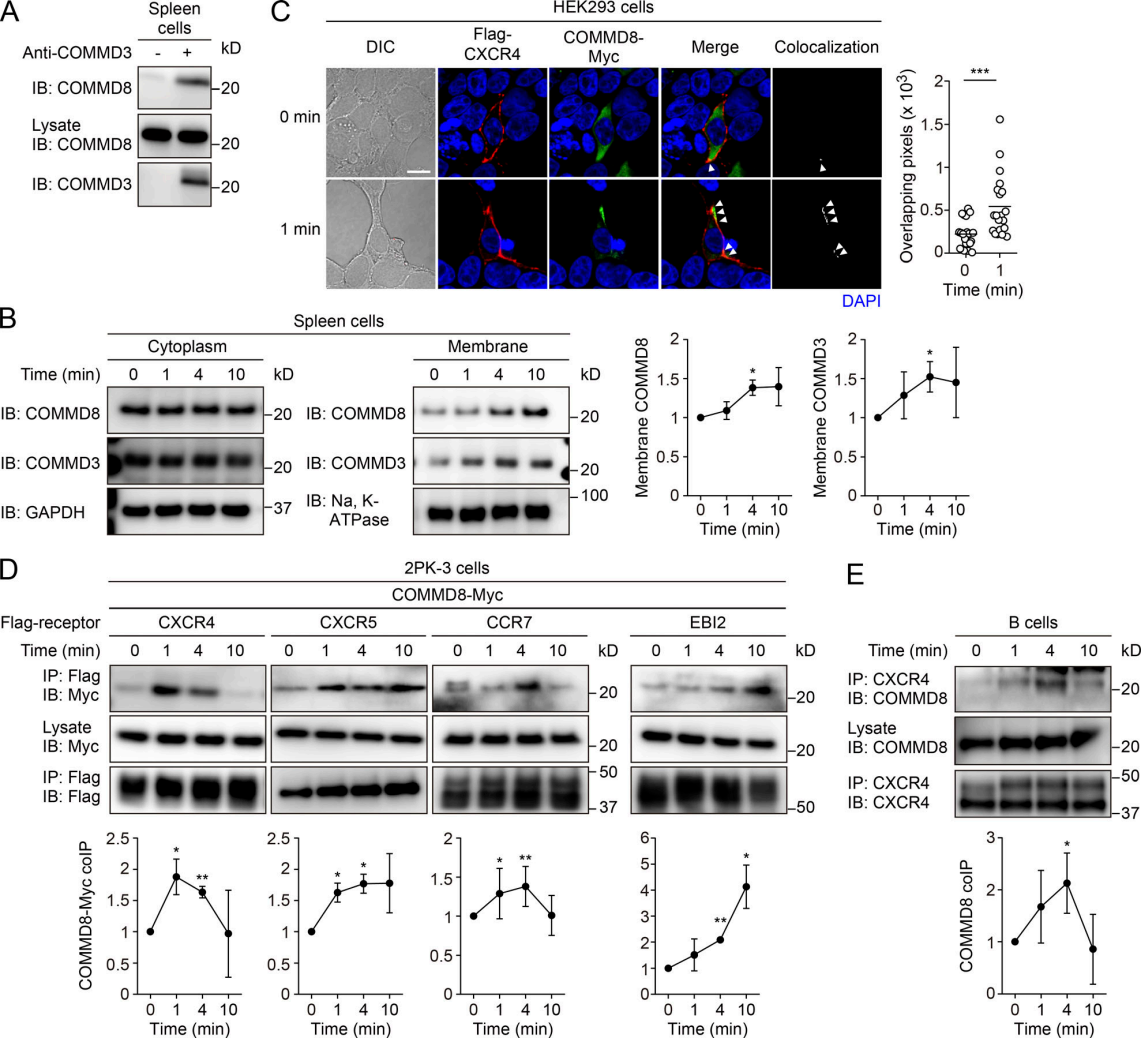
**COMMD8 forms a complex with COMMD3 and interacts with chemoattractant receptors. (A)** IP assay for the interaction between COMMD8 and COMMD3 in mouse spleen cells. **(B)** IB analysis for the membrane translocation of COMMD8 and COMMD3 in mouse spleen cells stimulated with CXCL12. **(C)** Confocal microscopy for the subcellular localization and colocalization of Flag-tagged CXCR4 (red) and Myc-tagged COMMD8 (green) in HEK293 cells before and at 1 min after CXCL12 treatment. Colocalization of the signals (arrowheads) was quantified by measurement of overlapping pixel numbers in each cell. Each symbol represents an individual cell, and bars indicate means (0 min, *n* = 20; 1 min, *n* = 20). Representative images are shown. Bar, 10 µm. **(D)** IP assay for the interaction of Myc-tagged COMMD8 with Flag-tagged CXCR4, CXCR5, CCR7, and EBI2 in 2PK-3 cells stimulated with their respective ligands: CXCL12, CXCL13, CCL19, and 7α,25-HC. **(E)** IP assay for the interaction of COMMD8 with CXCR4 in mouse primary B cells stimulated with CXCL12. Data are representative of three independent experiments (A) or pooled from three independent experiments (C). Error bars represent the mean ± SD of three (B and D) or four (E) independent experiments, and representative blots are shown. *, P < 0.05; **, P < 0.01; ***, P < 0.001. The P values were obtained by two-tailed paired *t* test in comparison with the protein levels at 0 min (B, D, and E) or two-tailed unpaired *t* test (C). coIP, coimmunoprecipitation; DIC, differential interference contrast.

In an immunoprecipitation (IP) assay using mouse spleen cells, endogenously expressed COMMD8 and COMMD3 were coprecipitated (Fig. 1 A), confirming their interaction in mammalian cells. The interaction between these proteins depended on their C-terminal regions encompassing the COMM domains (Fig. S1, A–C). After stimulation of mouse spleen cells with the CXCR4 ligand CXCL12, the levels of COMMD8 and COMMD3 proteins were increased in the membrane fraction of the cells (Fig. 1 B). Consistently, some COMMD8 and COMMD3 proteins were translocated from the cytoplasm to the cell membrane and colocalized with CXCR4 in CXCL12-stimulated human embryonic kidney (HEK) 293 cells (Fig. 1 C and Fig. S1 D). An IP assay in 2PK-3 mouse B lymphoma cells expressing epitope-tagged COMMD8 and CXCR4 demonstrated that COMMD8 was recruited to CXCR4 after ligand stimulation (Fig. 1 D). The interaction of COMMD8 with CXCR4 required the receptor C terminus (Fig. S1 E). The ligand-induced recruitment of COMMD8 to CXCR4 was also observed in primary mouse B cells (Fig. 1 E). Notably, COMMD8 was also recruited to other lymphocyte chemoattractant receptors, CXCR5, CCR7, and EBI2, after stimulation of transduced 2PK-3 cells with their respective ligands, CXCL13, CCL19, and 7α,25-dihydroxycholesterol (7α,25-HC; Fig. 1 D). However, S1P-induced COMMD8 recruitment to S1PR1 was barely detectable (Fig. S1 F). These findings suggest that COMMD8 and COMMD3 form a complex that is recruited to ligand-activated chemoattractant receptors. However, we cannot exclude a possible participation of other molecules in the COMMD3/8 complex.

### COMMD8 and COMMD3 are interdependent for stability

**Figure 2.**
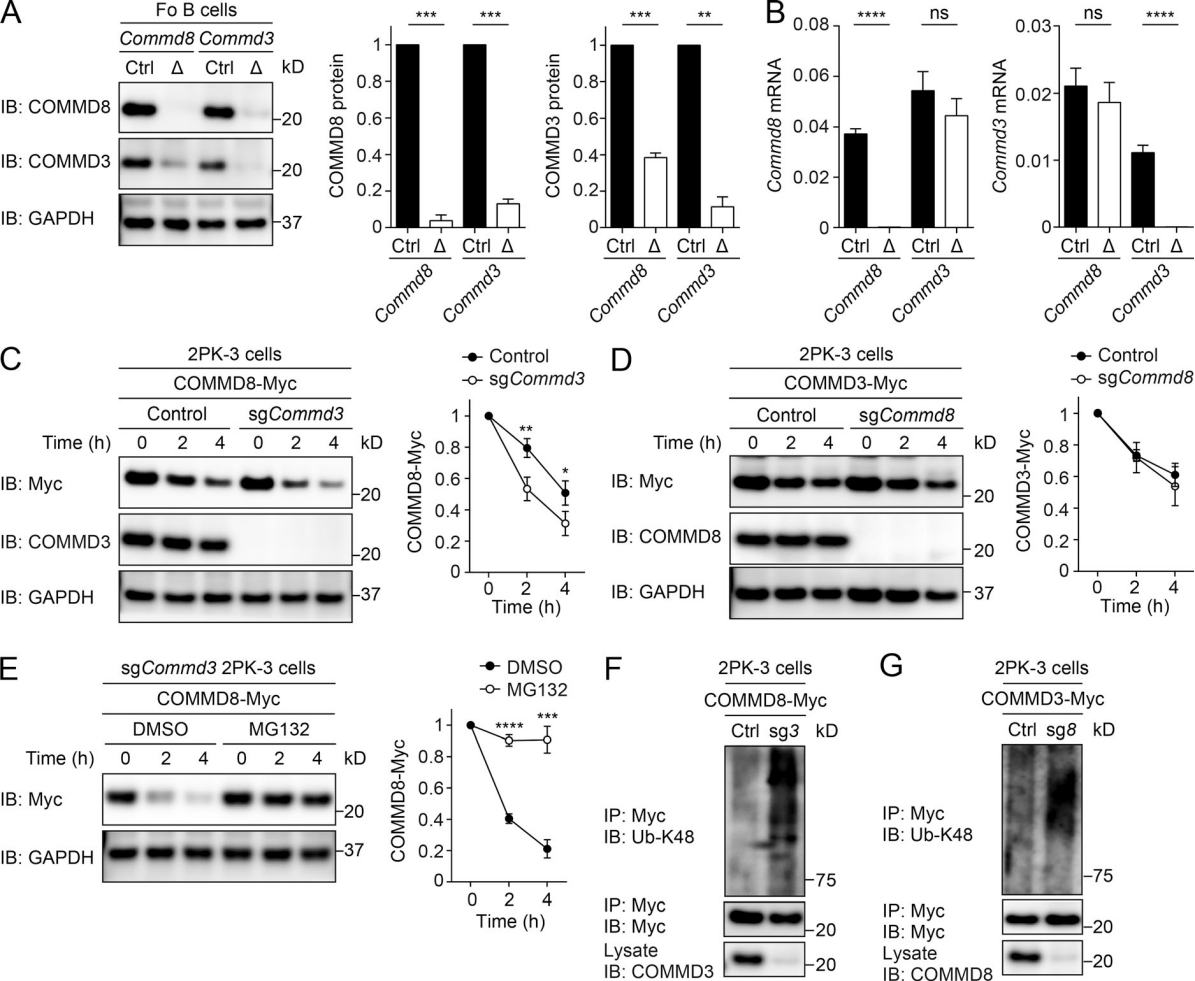
**COMMD8 and COMMD3 are interdependent for stability. (A)** IB analysis for the levels of COMMD8 and COMMD3 proteins in Fo B cells from *Commd8*Δ, *Commd3*Δ (Δ), and littermate control (Ctrl, *Commd8^f/+^Mb1^Cre/+^* or *Commd3^f/+^Mb1^Cre/+^*) mice. The COMMD8 and COMMD3 levels in mutant cells are expressed as the ratio to their levels in control cells and normalized to GAPDH levels. **(B)** Quantitative PCR analysis for the levels of *Commd8* (left) and *Commd3* (right) mRNAs relative to *Gapdh* expression in Fo B cells from *Commd8*Δ, *Commd3*Δ (Δ), and littermate control (Ctrl, *Commd8^f/+^Mb1^Cre/+^* or *Commd3^f/+^Mb1^Cre/+^*) mice. Data are shown as the mean + SD of triplicates and representative of three independent experiments. **(C and D)** IB analysis for the degradation of Myc-tagged COMMD8 (C) or COMMD3 (D) in vector-transfected control and COMMD3-deficient (sg*Commd3*; C) or COMMD8-deficient (sg*Commd8*; D) 2PK-3 cells at the indicated times after cycloheximide treatment. **(E)** IB analysis for the levels of Myc-tagged COMMD8 in sg*Commd3* 2PK-3 cells at the indicated times after treatment with MG132 or solvent DMSO in the presence of cycloheximide. **(F and G)** IB analysis for the K48-linked ubiquitination (Ub-K48) of Myc-tagged COMMD8 (F) or COMMD3 (G) in vector-transfected control (Ctrl) and sg*Commd3* (sg*3*; F) or sg*Commd8* (sg*8*; G) 2PK-3 cells. Data are representative of three independent experiments. Error bars represent the mean ± SD of three independent experiments, and representative blots are shown (A and C–E). *, P < 0.05; **, P < 0.01; ***, P < 0.001; ****, P < 0.0001; ns, not significant. The P values were obtained by two-tailed paired (A) or unpaired (B–E) *t* test.

To functionally characterize COMMD8 and COMMD3, we generated gene-targeted mice deficient in either of these proteins. Because mice lacking expression of *Commd8* or *Commd3* in the entire body were embryonic lethal, we deleted either of the genes using *Mb1^Cre/+^* mice that specifically express Cre recombinase in B cells (Hobeika et al., 2006). In an initial assessment of gene deletion, we unexpectedly found that, in follicular (Fo) B cells from *Commd3^f/f^Mb1^Cre/+^* (*Commd3*Δ) mice, COMMD8 protein was barely detectable despite normal levels of *Commd8* transcripts (Fig. 2, A and B). Substantial, but not complete, loss of COMMD3 protein was also observed in Fo B cells from *Commd8^f/f^Mb1^Cre/+^* (*Commd8*Δ) mice (Fig. 2 A). Deletion of *Commd3* using a single guide RNA in 2PK-3 cells accelerated degradation of COMMD8 in the presence of protein synthesis inhibitor cycloheximide (Fig. 2 C), whereas deletion of *Commd8* did not affect degradation of COMMD3 in this experimental setting (Fig. 2 D). Treatment of COMMD3-deficient cells with proteasome inhibitor MG132 restored the stability of COMMD8 for the experimental duration (4 h; Fig. 2 E), suggesting that COMMD8 is degraded by the proteasome. Indeed, K48-linked ubiquitination of COMMD8 and COMMD3 was enhanced in the absence of the other (Fig. 2, F and G). These findings suggest that COMMD8 and COMMD3 are interdependent for their stability, with COMMD8 having a stronger dependency. Consistently, transfection of COMMD8 in 2PK-3 cells elevated the level of endogenous COMMD3 (Fig. S1 G), probably because the stability of COMMD3 was increased by forming a complex with overexpressed COMMD8, which would in turn protect COMMD8 from degradation.

### Deficiency of the COMMD3/8 complex impairs B cell migration and humoral immune responses

**Figure 3.**
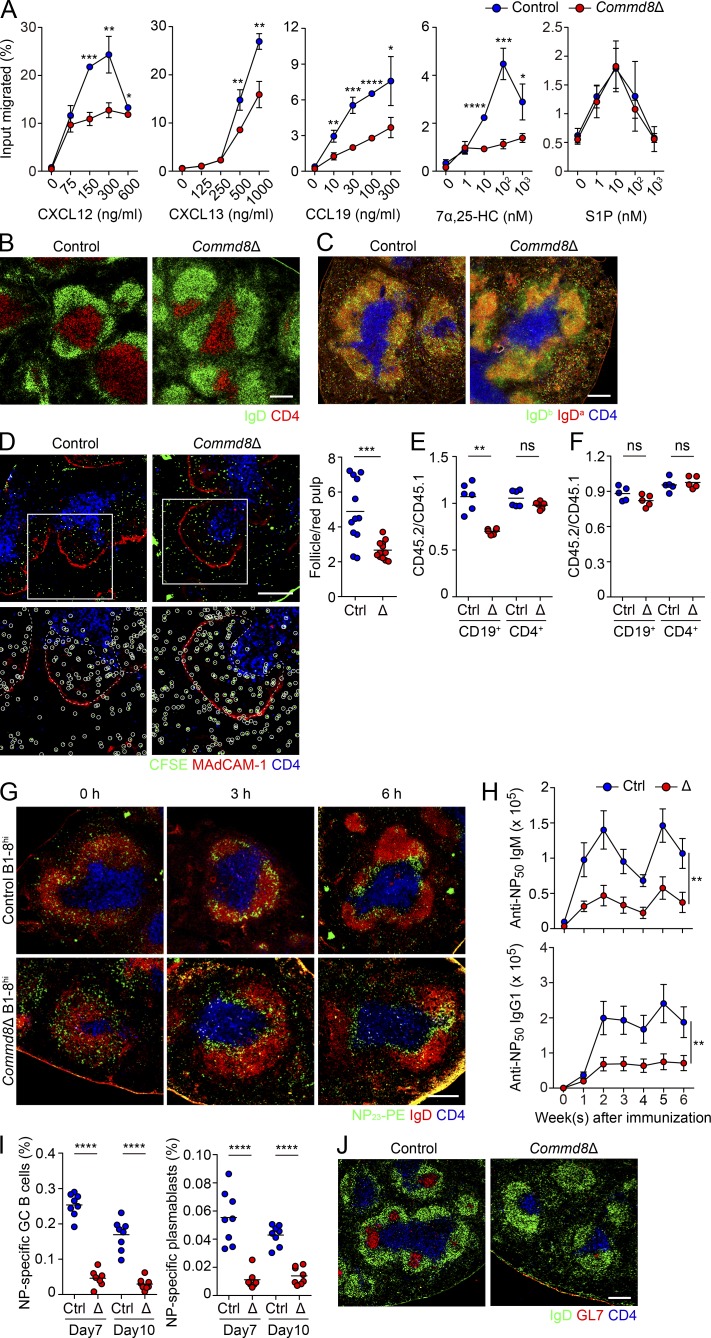
**COMMD8 deficiency impairs B cell migration and humoral immune responses. (A)** Chemotactic responses of control and *Commd8*Δ B cells were assessed by transwell migration of Fo B cells toward CXCL12, CXCL13, CCL19, 7α,25-HC, and S1P. Data are shown as the mean ± SD of triplicates. **(B)** Immunofluorescence microscopy of spleen tissues from control and *Commd8*Δ mice to detect B cells (IgD; green) and T cells (CD4; red). **(C)** Distribution of control and *Commd8*Δ B cells in the spleen of mixed bone marrow chimeras (20% Igh^b^ control or *Commd8*Δ plus 80% Igh^a^ WT). Sections were stained to detect Igh^b^ B cells (IgD^b^; green), Igh^a^ B cells (IgD^a^; red), and T cells (CD4; blue). **(D)** Control (Ctrl) and *Commd8*Δ (Δ) B cells were labeled with CFSE and transferred into WT mice. At 24 h after cell transfer, density of CFSE^+^ cells (green) in the follicles relative to the red pulp was assessed in the spleen sections stained for CD4 (blue) and MAdCAM-1 (red). The boxed areas are magnified to show CFSE^+^ cells (circled) and the perimeter of the follicles (dashed line). Each symbol represents an individual analyzed field, and bars indicate means (*n* = 12). **(E and F)** A mixture of CFSE-labeled LN cells (50% CD45.2 control or *Commd8*Δ plus 50% CD45.1 WT) were transferred into WT mice. LN entry of CD45.2 relative to CD45.1 B cells (CD19^+^) was assessed at 90 min after transfer (E). B cell egress from LNs was assessed at 12 h after blockade of LN entry (F). Transferred CD4^+^ T cells were tracked as an additional control. **(G)** Distribution of NP-binding B cells (green) in the spleen of control and *Commd8*Δ B1-8^hi^ mice was analyzed at the indicated times after NP-PE injection. **(H–J)** Control (Ctrl) and *Commd8*Δ (Δ) mice were immunized by i.p. injection of NP-CGG on days 0 and 28. Serum antibody titers (H, shown as the mean ± SEM of 10 mice) and the generation of GC B cells and plasmablasts in the spleen (I, shown as the percentages among B cells and total lymphocytes, respectively) were measured at the indicated times. Spleen sections were analyzed on day 7 to detect GCs (GL7; red), follicles (IgD; green), and T cell zones (CD4; blue; J). Littermate *Commd8^f/+^Mb1^Cre/+^* (C–E) or *Commd8^f/f^Mb1^+/+^* (A, B, and F–J) mice served as the control. Data are representative of three (A and G) or two (B, C, F, and J) independent experiments, or pooled from two (D and E) or three (H and I) independent experiments. Each symbol represents an individual mouse, and bars indicate means (E, F, and I). *, P < 0.05; **, P < 0.01; ***, P < 0.001; ****, P < 0.0001; ns, not significant. The P values were obtained by two-tailed unpaired (A, D–F, and I) or paired (H) *t* test. Bars, 200 µm.

To investigate the role of COMMD8 and COMMD3 in chemoattractant receptor signaling, we performed transwell migration assays for chemotactic responses of COMMD8- and COMMD3-deficient B cells. The mutant B cells exhibited reduced responses to CXCL12, CXCL13, CCL19, and 7α,25-HC compared with control B cells, while responses to S1P were unaffected (Fig. 3 A and Fig. S2, A and B). The mutant Fo B cells expressed normal levels of CXCR4, CXCR5, and EBI2, and rather higher levels of CCR7 on the cell surface (Fig. S2, C and D). Thus, the COMMD3/8 complex positively regulates signaling of chemoattractant receptors to which it is recruited.

To assess the effects of COMMD8 or COMMD3 deficiency on B cell migration in vivo, we performed histological analysis of lymphoid tissues from *Commd8*Δ and *Commd3*Δ mice and noticed less defined boundaries of B cell follicles in the spleen (Fig. 3 B and Fig. S2 E). In mixed bone marrow chimeras generated with COMMD8- or COMMD3-deficient and WT cells, the mutant B cells were localized beyond the follicular perimeter defined by WT B cells (Fig. 3 C and Fig. S2 F). Furthermore, transferred COMMD8- and COMMD3-deficient B cells were less confined to follicles than control B cells (Fig. 3 D and Fig. S2 G). These observations suggest impairment of CXCR5-mediated follicular localization of B cells by COMMD8 or COMMD3 deficiency. Consistent with the reduced CCR7- and CXCR4-mediated chemotaxis of COMMD8- and COMMD3-deficient B cells, COMMD8 or COMMD3 deficiency had negative effects on LN entry of B cells (Fig. 3 E and Fig. S2, H and J). As expected from the intact S1PR1 responsiveness of the mutant B cells, LN egress of B cells was not affected by COMMD8 or COMMD3 deficiency (Fig. 3 F and Fig. S2 I). These findings indicate that the COMMD3/8 complex controls multiple steps of homeostatic B cell trafficking.

Within a few hours of antigen encounter, EBI2 directs B cells to the outer follicle, close to the sites of antigen entry to lymphoid tissues, and promotes further antigen acquisition by the cells (Kelly et al., 2011). By 6 h after antigen encounter, CCR7 dominates over the EBI2-dependent outer follicle tropism and distributes activated B cells along the interface between the B cell follicle and T cell zone (B-T boundary; Kelly et al., 2011). To test whether deficiency of the COMMD3/8 complex affects B cell positioning during the early stages of activation, *Commd8*Δ mice harboring the 4-hydroxy-3-nitrophenylacetyl (NP)–specific B1-8^hi^ Ig heavy chain (*Commd8*Δ B1-8^hi^ mice) were immunized with NP-conjugated PE (NP-PE) to facilitate detection of NP-binding B cells by PE fluorescence. Consistent with the EBI2 unresponsiveness caused by deficiency of the COMMD3/8 complex, COMMD8-deficient B cells phenocopied EBI2-deficient B cells in their positioning during early activation (Kelly et al., 2011; Fig. 3 G). At 3 h after antigen injection, NP-binding B cells in control B1-8^hi^ mice were enriched in the outer follicle in the spleen, whereas those in *Commd8*Δ B1-8^hi^ mice failed to move to this region and had already arrived at the B-T boundary. At 6 h after NP-PE injection, antigen-activated B cells in both control and *Commd8*Δ B1-8^hi^ mice were distributed at the B-T boundary. The effect of partial reduction of CCR7 responsiveness by COMMD8 deficiency might be masked in the absence of EBI2-mediated attraction to the outer follicle, allowing activated B cells to access the B-T boundary. Since EBI2 deficiency in mice led to a reduction of antibody responses (Gatto et al., 2009; Pereira et al., 2009), the defect in EBI2-guided positioning of activated B cells prompted us to test the impacts of COMMD8 or COMMD3 deficiency on humoral immune responses. We found that *Commd8*Δ and *Commd3*Δ mice mounted reduced antibody responses after immunization with NP-conjugated chicken γ-globulin (NP-CGG), which was accompanied by impaired generation of germinal center (GC) B cells and plasmablasts (Fig. 3, H–J; and Fig. S2, K–N). These findings suggest that the COMMD3/8 complex plays an important role in the induction of humoral immune responses.

It was reported that RNA interference–mediated knockdown of COMMD8 in HEK293 cells retarded degradation of inhibitor of κB (IκB) after stimulation with TNF, suggesting that COMMD8 is a positive regulator of NF-κB signaling (Starokadomskyy et al., 2013). Indeed, COMMD8-deficient B cells showed slight retardation of IκB degradation after stimulation of B cell receptors (BCRs; Fig. S2 O). Additionally, a small reduction of BCR-induced intracellular calcium responses was observed in COMMD8-deficient B cells (Fig. S2 P). These observations suggest that the COMMD3/8 complex might be involved in BCR signaling. Although expression of *Commd8* and *Commd3* transcripts were detectable throughout B cell development (Fig. S3, A and B), the sizes of developing B cell subsets in the bone marrow were normal in *Commd8*Δ and *Commd3*Δ mice (Fig. S3, C and G). We speculate that the partial defects in CXCR4-mediated migration of COMMD8- and COMMD3-deficient B cells may be insufficient to reproduce the bone marrow phenotypes caused by complete loss of CXCR4 functions (Ma et al., 1999; Nie et al., 2004). However, there were reductions of mature and Fo B cells in the bone marrow, blood, and spleen of these mutant mice (Fig. S3, C–O). Considering the exceptionally high expression of *Commd8* in immature T1 B cells (Fig. S3 A), deficiency of the COMMD3/8 complex might have the greatest impact on B cells at this stage, which could impair BCR signaling and thereby transition from immature to mature B cells. Consistent with the reduction of mature B cells, serum levels of Ig isotypes, except for IgA, were decreased in *Commd8*Δ and *Commd3*Δ mice (Fig. S3, P and Q).

### The COMMD3/8 complex promotes β-arrestin–mediated signaling of CXCR4

**Figure 4.**
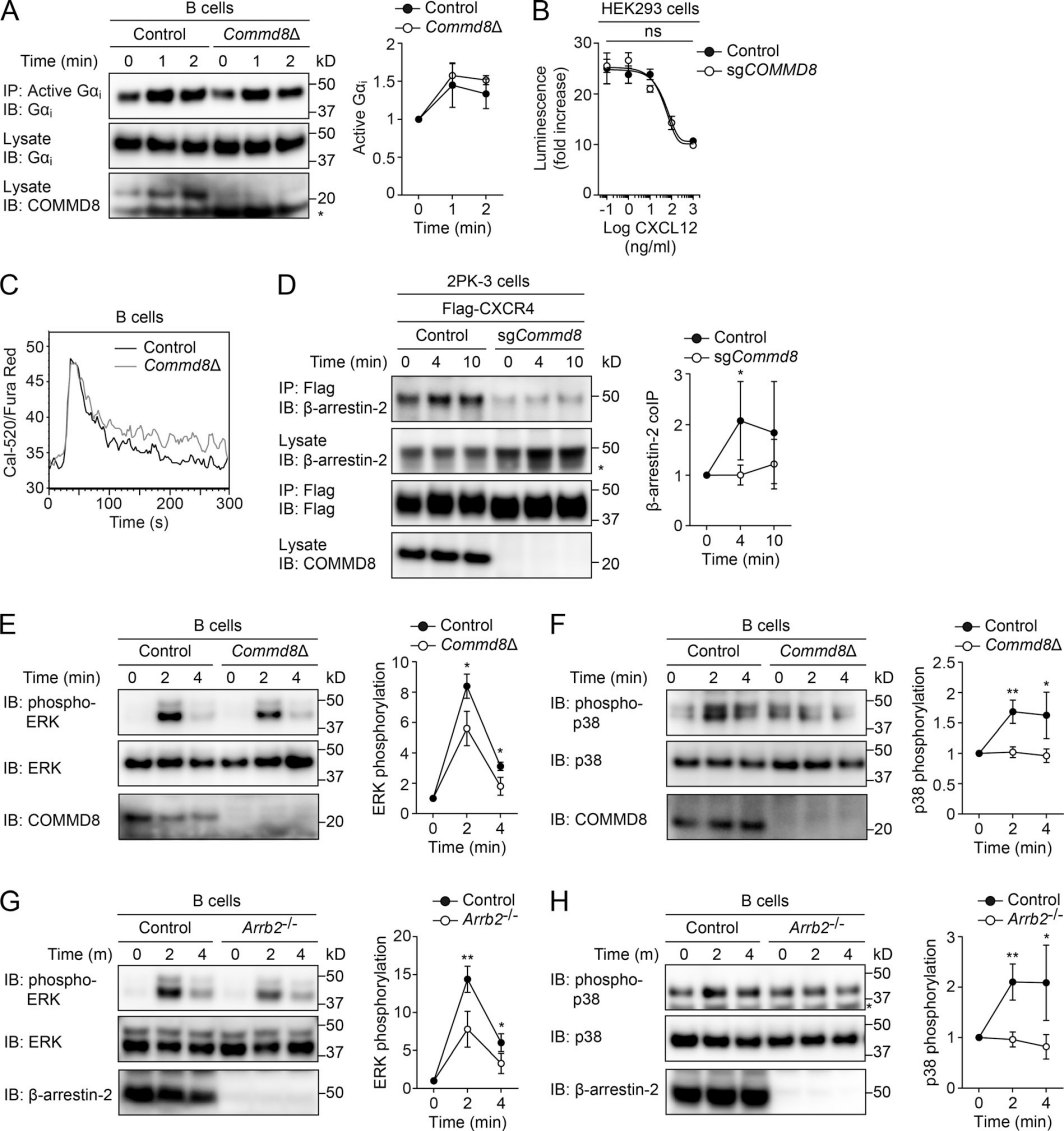
**COMMD8 promotes β-arrestin–mediated signaling of CXCR4. (A)** IB analysis for Gα_i_ activation induced by CXCL12 in control and *Commd8*Δ B cells. **(B)** CXCR4-mediated inhibition of cAMP production was assessed by the GloSensor reporter in vector-transfected control and COMMD8-deficient (sg*COMMD8*) HEK293 cells expressing Flag-tagged CXCR4. The amounts of cAMP are plotted as fold increases of luminescence over the levels in unstimulated cells. Data are shown as the mean ± SD of triplicates and representative of three independent experiments. **(C)** Intracellular calcium responses to CXCL12 in control and *Commd8*Δ B cells. Responses are plotted as the ratio of Cal-520 to Fura Red fluorescence. Data are representative of two independent experiments. **(D)** IP assay for the recruitment of endogenous β-arrestin-2 to Flag-tagged CXCR4 in vector-transfected control and COMMD8-deficient (sg*Commd8*) 2PK-3 cells after stimulation with CXCL12. **(E–H)** IB analysis for the phosphorylation of ERK (E and G) and p38 (F and H) in *Commd8*Δ (E and F) and *Arrb2^−/−^* (G and H) B cells after stimulation with CXCL12. B cells from littermate *Commd8^f/+^Mb1^Cre/+^* and *Commd8^f/f^Mb1^+/+^* (E and F) or *Arrb2^+/+^* (G and H) mice served as the control. Error bars represent the mean ± SD of three (A and E–H) or four (D) independent experiments, and representative blots are shown. *, P < 0.05; **, P < 0.01; ns, not significant. The P values were obtained by two-tailed unpaired (A and D–H) or paired (B) *t* test. Asterisk indicates nonspecific bands (A, D, and H). coIP, coimmunoprecipitation.

To elucidate the mechanism by which the COMMD3/8 complex promotes chemoattractant receptor signaling, we chose CXCR4 as a model because of the availability of reagents to analyze the downstream signaling. Agonist binding to chemoattractant receptors activates Gα_i_ proteins (Rot and von Andrian, 2004; Cyster et al., 2014; Griffith et al., 2014), which leads to suppression of cAMP production and induction of calcium mobilization. CXCL12-induced activation of Gα_i_ proteins was intact in B cells from *Commd8*Δ and *Commd3*Δ mice (Fig. 4 A and Fig. S4 A). Deletion of *COMMD8* or *COMMD3* in HEK293 cells did not affect CXCR4-mediated suppression of cAMP production, which was assessed by a live cell cAMP biosensor, GloSensor (Fan et al., 2008; Fig. 4 B and Fig. S4 B). Moreover, CXCR4-medited intracellular calcium responses were not impaired in COMMD8- and COMMD3-deficient B cells (Fig. 4 C and Fig. S4 C). These observations suggest that the COMMD3/8 complex is not involved in Gα_i_ activation through CXCR4.

Next, we examined the role of the COMMD3/8 complex in β-arrestin–mediated signaling of CXCR4. Deletion of *Commd8* or *Commd3* in 2PK-3 cells reduced the recruitment of endogenous β-arrestin-2 (also known as arrestin-3) to epitope-tagged CXCR4 (Fig. 4 D and Fig. S4 D). COMMD8- and COMMD3-deficient B cells showed reduced phosphorylation of ERK and p38 at 2 and 4 min after stimulation with CXCL12 (Fig. 4, E and F; and Fig. S4, E and F). B cells from β-arrestin-2–deficient mice also exhibited defects in the activation of these MAPKs at the same time points (Fig. 4, G and H), indicating that β-arrestin-2 is involved in the MAPK activation at these times. Because COMMD8- and COMMD3-deficient B cells expressed the comparable levels of β-arrestin-2 with the control cells (Fig. S5 D), the defects of MAPK activation in the mutant cells were not due to low expression of β-arrestin-2. However, B cells deficient in β-arrestin-1 (also known as arrestin-2) showed no defects in MAPK activation after CXCR4 stimulation (Fig. S4, G and H), negating the contribution of β-arrestin-1 to CXCR4-mediated MAPK activation in B cells. Therefore, the reduction of CXCR4-mediated MAPK activation in COMMD8- and COMMD3-deficient B cells might be, at least in part, attributable to the impaired recruitment of β-arrestin-2. Using inhibitors for ERK and p38, we confirmed the involvement of these MAPKs in CXCR4-mediated migration of B cells (Fig. S4, I and J). These findings suggest that the COMMD3/8 complex promotes β-arrestin–mediated MAPK activation downstream of CXCR4, which may enhance chemotactic responses of B cells. In support of this notion, a study has shown that β-arrestin-2 deficiency impairs CXCR4-mediated chemotaxis of B cells to a similar extent as COMMD8 or COMMD3 deficiency (Fong et al., 2002).

Unexpectedly, however, COMMD8 or COMMD3 deficiency in Fo B cells produced minimal effects on ligand-induced internalization of CXCR4 (Fig. S4, K and L). Surprisingly, the internalization of CXCR4 was intact in B cells deficient in both β-arrestin-1 and β-arrestin-2 (Fig. S4 M). Therefore, contrary to the established roles of β-arrestins in GPCR internalization, CXCR4 internalization in B cells occurs independently of β-arrestins, which may account for the intact CXCR4 internalization in COMMD8- and COMMD3-deficient B cells. It has been shown that a clathrin adaptor AP2 acts independently of β-arrestins to promote internalization of chemokine receptors (Marchese, 2014). Such a β-arrestin–independent mechanism might play a dominant role in CXCR4 internalization in B cells.

### The COMMD3/8 complex is required for GRK6-mediated phosphorylation of CXCR4

**Figure 5.**
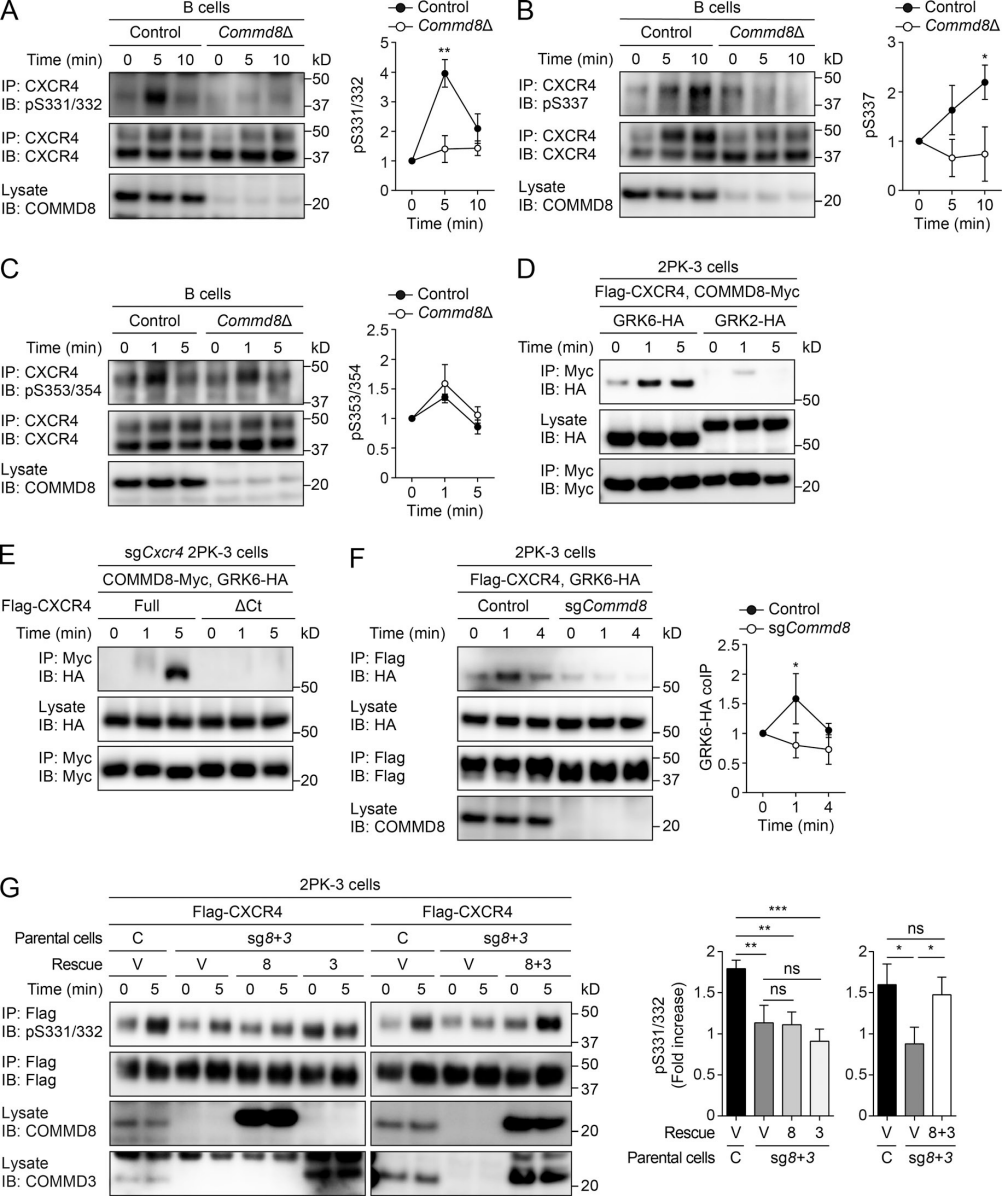
**COMMD8 is required for GRK6-mediated phosphorylation of CXCR4. (A–C)** IB analysis for the phosphorylation (p) at S331/332 (A), S337 (B), and S353/354 (C) of CXCR4 in control (*Commd8^f/f^Mb1^+/+^*) and *Commd8*Δ B cells after stimulation with CXCL12. **(D)** IP assay for the interaction of Myc-tagged COMMD8 with HA-tagged GRK6 or GRK2 in 2PK-3 cells expressing Flag-tagged CXCR4 after stimulation with CXCL12. Data are representative of three independent experiments. **(E)** IP assay for the interaction of Myc-tagged COMMD8 and HA-tagged GRK6 in CXCR4-deficient (sg*Cxcr4*) 2PK-3 cells expressing a C terminus–truncated mutant of Flag-tagged CXCR4 (ΔCt) after stimulation with CXCL12. Flag-tagged full-length CXCR4 (Full) served as a positive control. Data are representative of two independent experiments. **(F)** IP assay for the interaction of Flag-tagged CXCR4 with HA-tagged GRK6 in vector-transfected control and COMMD8-deficient (sg*Commd8*) 2PK-3 cells stimulated with CXCL12. **(G)** CXCL12-induced S331/332 phosphorylation of CXCR4 in 2PK-3 cells doubly deficient of COMMD8 and COMMD3 (sg*8*+*3*) after rescuing either (8 or 3, left) or both (8+3, right) of them. Data are shown as fold increases of S331/332 phosphorylation over unstimulated (0 min) samples. C, control; V, vector. Error bars represent the mean ± SD of three independent experiments, and representative blots are shown (A–C, F, and G). *, P < 0.05; **, P < 0.01; ***, P < 0.001; ns, not significant. The P values were obtained by two-tailed unpaired *t* test (A–C and F) or one-way ANOVA with Tukey’s post hoc test (G). coIP, coimmunoprecipitation.

Studies of several GPCRs, including CXCR4, revealed that site-specific phosphorylation by different GRKs induces distinct signaling, which led to the consensus that phosphorylation by GRK6 or GRK5 promotes β-arrestin–mediated MAPK activation, whereas phosphorylation by GRK2 or GRK3 opposes it (Kim et al., 2005; Ren et al., 2005; Busillo et al., 2010; Butcher et al., 2011; Nobles et al., 2011). Using antibodies that recognize specific phosphorylated residues on the C terminus of CXCR4 (Busillo et al., 2010; Mueller et al., 2013), we found that GRK6-mediated phosphorylation at S331/332 and S337 was abolished in B cells from *Commd8*Δ and *Commd3*Δ mice (Fig. 5, A and B; and Fig. S5, A and B), while S353/354 phosphorylation mediated by GRK2 and GRK3 remained intact (Fig. 5 C and Fig. S5 C). We confirmed that COMMD8- and COMMD3-deficient B cells expressed the comparable levels of GRK6 with the control cells (Fig. S5 D). IP assays in 2PK-3 cells showed that COMMD8 and COMMD3 interacted with GRK6, but not GRK2 or GRK5, after CXCR4 stimulation (Fig. 5 D and Fig. S5, E and F). The interaction between COMMD8 and GRK6 was not induced in 2PK-3 cells expressing a CXCR4 mutant lacking the C-terminal tail (Fig. 5 E), suggesting that the interaction occurs on the CXCR4 tail. Deletion of *Commd8* or *Commd3* in 2PK-3 cells reduced GRK6 recruitment to CXCR4 (Fig. 5 F and Fig. S5 G). However, GRK6 deficiency in 2PK-3 cells had little effect on COMMD8 recruitment to CXCR4 (Fig. S5 H). Therefore, GRK6 recruitment to CXCR4 depends on the COMMD3/8 complex, but recruitment of the COMMD3/8 complex to the receptor occurs independently of GRK6. In 2PK-3 cells that were deficient in both *Commd8* and *Commd3*, rescuing both COMMD8 and COMMD3 restored GRK6-mediated phosphorylation of CXCR4, but rescuing either of them did not (Fig. 5 G). Thus, both COMMD proteins are required for the function of their complex, which may account for the phenocopy of COMMD8 and COMMD3 deficiency. These findings suggest that the COMMD3/8 complex recruits GRK6, but not other lymphocyte GRKs, to CXCR4 and promotes site-specific phosphorylation of the receptor C terminus. Because previous studies demonstrated that S331/332 of CXCR4 were also phosphorylated by protein kinase C (Busillo et al., 2010; Luo et al., 2017), we cannot exclude a possibility that the COMMD3/8 complex might play a role in the recruitment of this kinase to CXCR4.

### Recruitment of the COMMD3/8 complex to CXCR4 depends on GRK2-mediated phosphorylation

**Figure 6.**
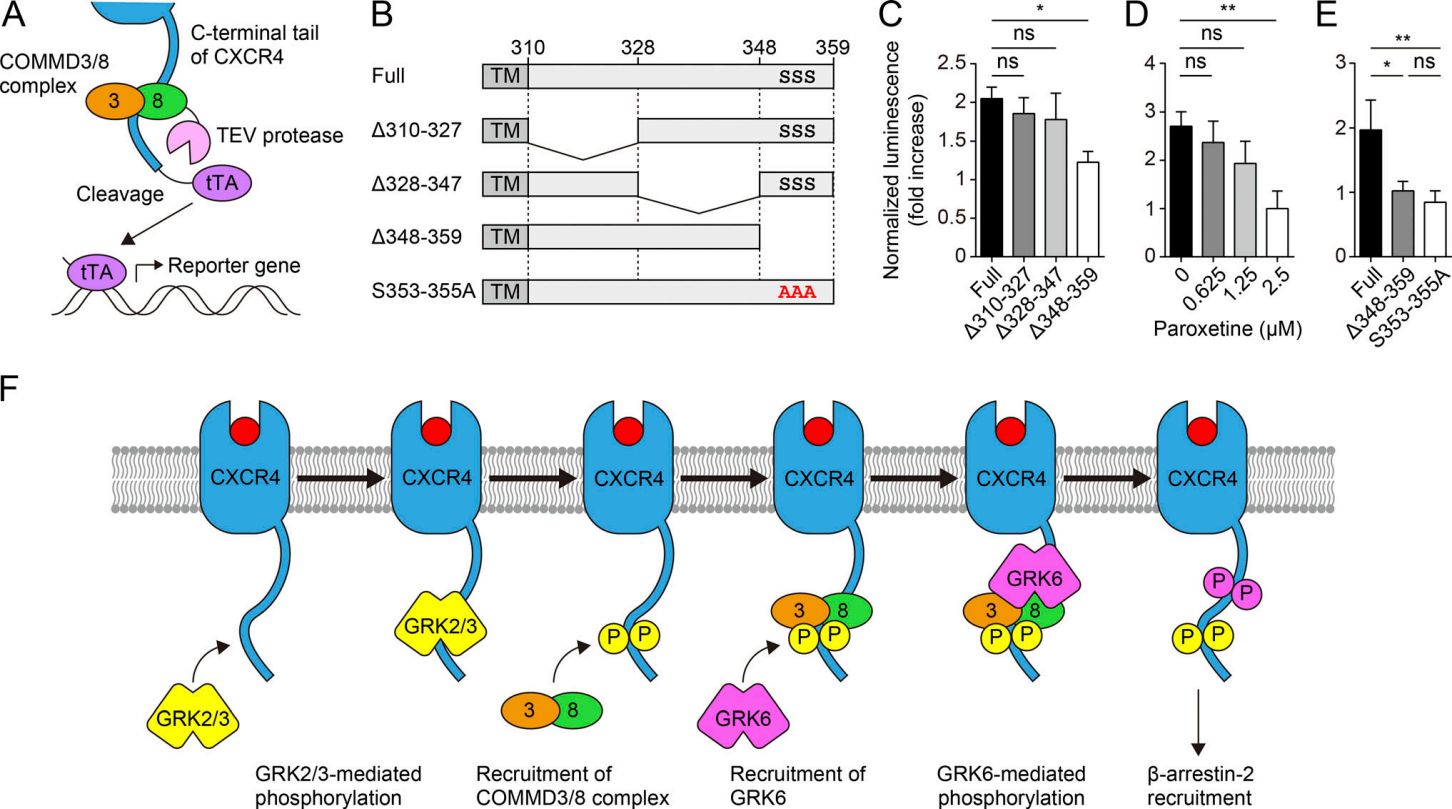
**Recruitment of the COMMD3/8 complex to CXCR4 depends on GRK2-mediated phosphorylation. (A)** Schematic diagram of the Tango assay for detecting the association of COMMD8 with CXCR4 in HTL cells. **(B)** Diagrams of the C termini of the mouse CXCR4 mutants used in C and E. **(C)** Tango assay for the association of COMMD8 with C-terminal deletion mutants of CXCR4. **(D)** Tango assay for the association of COMMD8 and WT CXCR4 in the presence of GRK2 inhibitor paroxetine. **(E)** Tango assay for the association of COMMD8 with a CXCR4 mutant harboring alanine substitution of GRK2 phosphorylation sites (S353-355A). The Δ348-359 mutant served as a negative control. **(F)** Proposed role of the COMMD3/8 complex in GRK6 recruitment to CXCR4. The luminescence from the tTA-dependent firefly luciferase reporter was normalized to that from the cotransfected Renilla luciferase and plotted as fold increases over the levels in unstimulated cells (D–F). Data are shown as the mean ± SD of triplicates and representative of three experiments. *, P < 0.05; **, P < 0.01; ns, not significant. The P values were obtained by one-way ANOVA with Tukey’s post hoc test (C and E) or two-tailed unpaired *t* test (D). TM, transmembrane domain.

To clarify how the COMMD3/8 complex interact with CXCR4, we first sought to determine which region of the CXCR4 C-terminal tail is responsible for the interaction with the COMMD3/8 complex. To facilitate the analysis of the interaction, we employed the Tango assay (Barnea et al., 2008). Specifically, we fused COMMD8 to the N terminus of tobacco etch virus (TEV) protease, and GPCRs via a TEV protease cleavage site to a tetracycline-controlled transcriptional activator (tTA). These proteins were expressed in HTL cells, a HEK293T-derived cell line containing a stably integrated tTA-dependent firefly luciferase reporter gene. Close association between COMMD8 and the GPCR would bring the TEV protease in the direct vicinity of the cleavage site to cut and release tTA, which allows tTA to enter the nucleus and activate the expression of the luciferase reporter (Fig. 6 A). In Tango assays for the association of COMMD8 with C-terminal deletion mutants of CXCR4, we found that the extreme end of the C-terminal tail was required for the association (Fig. 6, B and C). Because this region contains the phosphorylation sites of GRK2 (S353/354; Mueller et al., 2013), we next examined whether the association of the COMMD3/8 complex with CXCR4 is linked to GRK2 activity. Paroxetine, a selective serotonin reuptake inhibitor recently shown to also inhibit GRK2 activity (Thal et al., 2012; Schumacher et al., 2015), reduced the association between CXCR4 and COMMD8 in a dose-dependent manner (Fig. 6 D). Additionally, alanine substitution of S353/354/355 abrogated the association (Fig. 6 E). These findings suggest that the interaction of the COMMD3/8 complex with CXCR4 depends on GRK2-mediated phosphorylation of the receptor C terminus. Because GRK3 also phosphorylates S353/354 of CXCR4 (Mueller et al., 2013), GRK3 activity may contribute to the interaction. The functional redundancy between GRK2 and GRK3 may account for the fact that in contrast to COMMD8 or COMMD3 deficiency, GRK2 deficiency causes no significant defects in CXCR4-mediated migration of B cells (Arnon et al., 2011).

The Tango assay suggests that the COMMD3/8 complex comes in close proximity to and might directly interact with the C terminus of ligand-activated CXCR4. Therefore, the association between CXCR4 and the COMMD3/8 complex might be maintained by electrostatic interactions between the phosphorylated residues of CXCR4 and positively charged sites on the surface of the COMMD3/8 complex. As shown above, recruitment of the COMMD3/8 complex to CXCR4 occurred independently of GRK6 (Fig. S5 H), and the COMMD3/8 complex interacted with GRK6 on the C-terminal tail of CXCR4 (Fig. 5 E). Taken together with these findings, we propose a stepwise mechanism for GRK6 recruitment to CXCR4 (Fig. 6 F). First, the C terminus of activated CXCR4 is phosphorylated by GRK2 and GRK3. Next, the COMMD3/8 complex is associated with the receptor C terminus through electrostatic interactions with the phosphorylated residues. Finally, GRK6 is recruited to the receptor through the interaction with the COMMD3/8 complex and phosphorylates the C terminus. This putative mechanism is compatible with a previous observation that CXCR4 phosphorylation by GRK2 and GRK3 preceded phosphorylation by GRK6 (Mueller et al., 2013).

### The COMMD3/8 complex is essential for GRK6-mediated phosphorylation of β_2_AR

**Figure 7.**
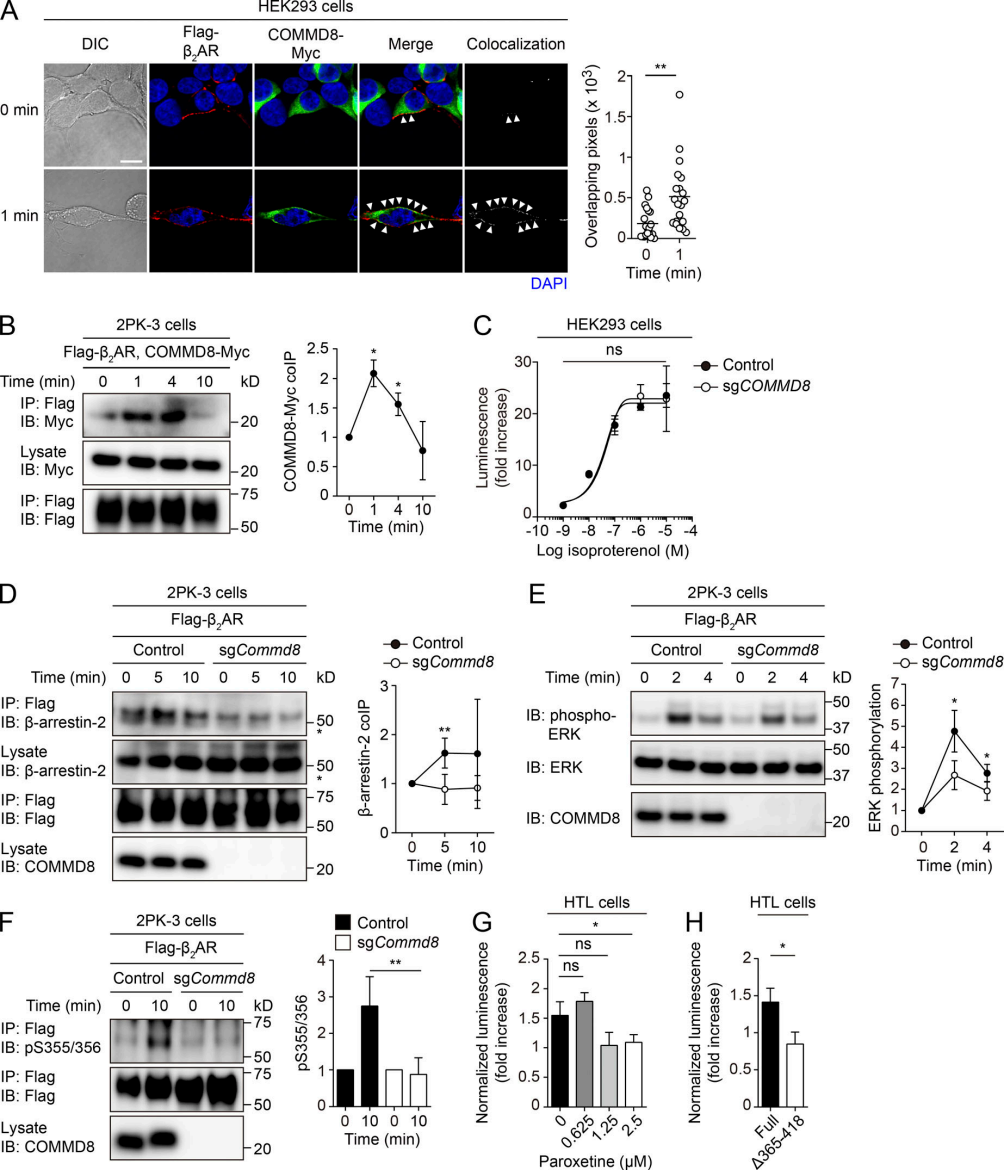
**COMMD8 is involved in β-arrestin-mediated signaling of β_2_AR. (A)** Confocal microscopy for the subcellular localization and colocalization of Flagg-tagged β_2_AR (red) and Myc-tagged COMMD8 (green) in HEK293 cells before and at 1 min after treatment with pan-βAR agonist isoproterenol. Colocalization of the signals (arrowheads) was quantified as in Fig. 1 C. Each symbol represents an individual cell, and bars indicate means (0 min, *n* = 20; 1 min, *n* = 20). Representative images are shown. Bar, 10 µm. **(B)** IP assay for the interaction of Myc-tagged COMMD8 with Flag-tagged β_2_AR in 2PK-3 cells after stimulation with selective β_2_AR agonist clenbuterol. **(C)** cAMP production assessed by GloSensor in vector-transfected control and COMMD8-deficient (sg*COMMD8*) HEK293 cells after stimulation with isoproterenol. The amounts of cAMP are plotted as fold increases of luminescence over the levels in unstimulated cells. Data are shown as the mean ± SD of triplicates and representative of three experiments. **(D)** IP assay for the recruitment of endogenous β-arrestin-2 to Flag-tagged β_2_AR in vector-transfected control and COMMD8-deficient (sg*Commd8*) 2PK-3 cells after stimulation with isoproterenol. Asterisks indicate nonspecific bands. **(E)** IB analysis for the phosphorylation of ERK in vector-transfected control and sg*Commd8* 2PK-3 cells after stimulation with isoproterenol. **(F)** IB analysis for GRK6-mediated phosphorylation (p) at S355/356 of Flag-tagged β_2_AR in vector-transfected control and sg*Commd8* 2PK-3 cells after stimulation with isoproterenol. Error bars represent the mean ± SD of three (B), four (E and F), or five (D) independent experiments, and representative blots are shown. **(G)** Tango assay for the association of COMMD8 with β_2_AR in the presence of paroxetine. **(H)** Tango assay for the association of COMMD8 with a C terminus–truncated β_2_AR mutant lacking GRK2 phosphorylation sites. The luminescence from the tTA-dependent firefly luciferase reporter was normalized to that from the cotransfected Renilla luciferase and plotted as fold increases over the levels in unstimulated cells (G and H). *, P < 0.05; **, P < 0.01; ns, not significant. The P values were obtained by two-tailed unpaired (A and D–H) or paired (B and C) *t* test. coIP, coimmunoprecipitation; DIC, differential interference contrast.

The interaction of the COMMD3/8 complex with multiple chemoattractant receptors suggested that it might also mediate signaling of other GPCR subfamilies. To test this possibility, we examined involvement of the COMMD3/8 complex in signaling of β_2_AR, the prototypical GPCR. Confocal microscopy for the subcellular localization of epitope-tagged COMMD8 and β_2_AR in HEK293 cells showed that COMMD8 was translocated to the cell membrane and colocalized with β_2_AR after agonist stimulation (Fig. 7 A). An IP assay in 2PK-3 cells demonstrated an agonist-induced interaction of β_2_AR with COMMD8 (Fig. 7 B). β_2_AR primarily transmits signals through Gα_s_, the activation of which promotes production of cAMP by stimulating adenylyl cyclase (Rosenbaum et al., 2009). The β_2_AR-mediated cAMP production was intact in COMMD8-deficient HEK293 cells (Fig. 7 C), suggesting that the COMMD3/8 complex is not involved in Gα_s_ activation through β_2_AR. However, the recruitment of β-arrestin-2 to agonist-activated β_2_AR was reduced in COMMD8-deficient 2PK-3 cells (Fig. 7 D). Consistently, COMMD8-deficient 2PK-3 cells showed a defect in ERK activation downstream of β_2_AR (Fig. 7 E). Additionally, GRK6-mediated phosphorylation at S355/356 of β_2_AR was abrogated by COMMD8 deficiency (Fig. 7 F). As in the case of CXCR4, COMMD8 recruitment to β_2_AR depended on GRK2 activity (Fig. 7 G). Indeed, deletion of the β_2_AR C-terminal region encompassing the reported GRK2 phosphorylation sites (Nobles et al., 2011) abrogated the association with COMMD8 (Fig. 7 H). In line with the role of the COMMD3/8 complex in chemoattractant receptor signaling, these findings suggest that this protein complex recruits GRK6 to β_2_AR in a GRK2-dependent manner and promotes β-arrestin–mediated MAPK activation. Thus, the COMMD3/8 complex might be involved in the initiation of β-arrestin–mediated signaling of a wide range of GPCRs by selectively recruiting GRK6.

## Discussion

It has been suggested that the specificity of GRK recruitment to GPCRs is determined by the relative expression levels of individual GRKs, which vary among cell types (Violin et al., 2006; Tobin et al., 2008), and distinct receptor conformations induced by ligand binding (Nobles et al., 2011). Our study identifies a GRK-recruiting adaptor, the COMMD3/8 complex, as an additional determinant of GRK specificity for GPCRs. Analysis of CXCR4 signaling suggests that the COMMD3/8 complex selectively recruits GRK6 to chemoattractant receptors in a GRK2-dependent manner, which triggers the MAPK pathway of β-arrestin–mediated signaling. Investigating the role of the COMMD3/8 complex in B cells reveals that this protein complex promotes their chemotactic responses, which is reflected in their migration in vivo and humoral immune responses. Therefore, β-arrestin–mediated chemoattractant receptor signaling initiated by the COMMD3/8 complex is important for proper functioning of the immune system.

B cell–specific *Commd8*- and *Commd3*-deficient mice exhibit compromised antibody responses with diminished production of GC B cells and plasmablasts. Although deficiency of the COMMD3/8 complex causes the same defect in positioning of early-activated B cells as EBI2 deficiency, the defects in humoral immune responses caused by B cell–specific deficiency of the COMMD3/8 complex are more severe than those observed in EBI2-deficient mice (Gatto et al., 2009; Pereira et al., 2009). This suggests that deficiency of the COMMD3/8 complex affects additional aspects of humoral immunity. Because CXCR5-mediated follicular localization is essential for antigen encounter by rare antigen-specific B cells (Junt et al., 2005; Suzuki et al., 2009), the defective follicular homing of *Commd8*- and *Commd3*-deficient B cells could decrease their chance of antigen recognition and consequently impair humoral immune responses. Additionally, deficiency of the COMMD3/8 complex causes slight impairment of BCR signaling and significant reduction of mature B cells, which is consistent with the fact that genetic deficiency in the components of chemoattractant receptor signaling results in measurable defects in BCR signaling and thereby B cell development (Walmsley et al., 2003; Henderson et al., 2010; Hwang et al., 2013; Boularan et al., 2015). The deficits in BCR signaling and mature B cell numbers may also contribute to the reduced humoral immune responses in the mutant mice.

The present study suggests that the COMMD3/8 complex may be associated with a range of GPCRs through electrostatic interactions with phosphorylated residues on the receptor C termini. However, the common motif recognized by the COMMD3/8 complex remains to be determined. A recent study demonstrated that a set of phosphorylation codes in the C-terminal tails of GPCRs is used for high-affinity binding of arrestins to the receptors (Zhou et al., 2017). The code is predicted to be *P*x*P*xx*P*/E/D or *P*xx*P*xx*P*/E/D, where *P* represents a phospho-serine or phospho-threonine and x represents any amino acid residue. An extensive network of electrostatic interactions is formed between the positively charged pockets of arrestin surface and the simple set of phosphorylated residues, which allows arrestins to interact with the highly divergent sequences of the GPCR tails. Therefore, it is conceivable that the COMMD3/8 complex might also recognize a motif that has minimal structural requirements and permits high variability in the amino acid sequence, which might account for less selective interactions of the COMMD3/8 complex with GPCRs.

COMMD proteins have been shown to interact with other proteins and participate in multimeric protein complexes such as Cullin-containing ubiquitin ligases (Maine et al., 2007; Mao et al., 2011) and the COMMD/CCDC22/CCDC93 complex (Li et al., 2015; Phillips-Krawczak et al., 2015; Bartuzi et al., 2016; McNally et al., 2017). Therefore, it is possible that COMMD8 and COMMD3 might be components of a large protein assembly. However, our results indicate that the interaction between the two COMMD proteins is critical for their stability and functions. By exploiting this unique property of the COMMD3/8 complex, it would be possible to degrade and disable the protein complex by pharmacological disruption of the interaction between COMMD8 and COMMD3. Development of such drugs would provide an approach to tightly control GPCR phosphorylation by GRKs for regulation of immune responses and other biological processes. As GRK6 is selectively recruited to GPCRs by the COMMD3/8 complex, other GRKs might also have specific adaptors for their recruitment to GPCRs. Identifying new GRK-recruiting adaptors would facilitate understanding of the molecular mechanism that generates distinct phosphorylation patterns in the C termini of GPCRs, which dictate the functional outcomes of β-arrestin–mediated signaling.

## Materials and methods

### Mice

C57BL/6 (B6) mice were purchased from Clea. Igh^a^ mice on the B6 background (RBRC01201) were obtained from the RIKEN BioResource Center. *Mb1^Cre/+^* mice on the B6 background (Hobeika et al., 2006) were provided by M. Reth (Max Planck Institute of Immunology and Epigenetics, Freiburg, Germany). *CAG^FLPe/+^* mice on the B6 background (Rodríguez et al., 2000) were provided by A.F. Stewart (Biotechnology Center, Technische Universität Dresden, Dresden, Germany). B1-8^hi^ mice on the B6 background (Shih et al., 2002) were generated by M.C. Nussenzweig (The Rockefeller University, New York, NY) and provided through T. Kurosaki (Osaka University, Osaka, Japan). *Arrb2^−/−^* mice on the B6 background (Arrb2*^tm1Rjl^*, MGI: 2157960) were purchased from The Jackson Laboratory. *Arrb1^f/f^* mice on the B6 background were generated from embryonic stem cells purchased from the Knockout Mouse Project Repository (*Arrb1^tm1a(KOMP)Wtsi^*, MGI: 99473) and bred with *Mb1^Cre/+^* mice to obtain *Arrb1^f/f^Mb1^Cre/+^* mice. *Arrb1^f/f^Arrb2^−/−^Mb1^Cre/+^* mice were generated by crossing *Arrb2^−/−^* mice with *Arrb1^f/f^Mb1^Cre/+^* mice. To generate *Commd8^f/f^* mice, exons 2–4 of *Commd8* were targeted by flanking them with *loxP* sites along with a *FRT*-flanked neomycin resistance cassette. The targeting vector for generation of *Commd3^f/f^* mice (PG00187_Z_7_H01), in which exons 2–7 of *Commd3* were flanked with *loxP* sites, was purchased from the European Mouse Mutant Cell Repository. The targeting vectors were transfected into EGR-G101 (Fujihara et al., 2013), an embryonic stem cell line of the B6 background, and homologous recombination was confirmed by PCR. Heterozygous *Commd8*- and *Commd3*-targeted mice were bred with *CAG^FLPe/+^* mice to delete the neomycin resistance cassette. The resulting *Commd8^f/f^* and *Commd3^f/f^* mice were intercrossed with *Mb1^Cre/+^* mice to excise the *loxP*-flanked allele. *Commd8*Δ B1-8^hi^ mice were generated by crossing *Commd8*Δ mice with B1-8^hi^ mice. Mice were mixed sexes and 8–12 wk old at the time of experiments. Mice within experiments were from littermates of the same sex. Bone marrow chimeras were generated by irradiating recipient mice with a single dose of 8 Gy, followed by i.v. transfer of 5–10 × 10^6^ bone marrow cells per mouse. Chimeras were analyzed ≥8 wk after bone marrow reconstitution. Mice were housed in a specific pathogen–free facility. All experiments were performed in accordance with protocols approved by the Osaka University Animal Care Committee.

### Cell lines

HEK293 cells (EC85120602; European Collection of Authenticated Cell Cultures) and their derivative Phoenix-E packaging cells, which were established in the laboratory of G.P. Nolan (Stanford University, Stanford, CA) and provided through J.G. Cyster (University of California, San Francisco, San Francisco, CA), were maintained in DMEM (Wako) supplemented with 10% FBS (Gibco), 2 mM L-glutamine (Wako), and 100 U/ml penicillin/streptomycin (Wako). 2PK-3 cells (TIB-203; American Type Culture Collection) were maintained in DMEM supplemented with 10% FBS, 2 mM L-glutamine, 55 µM 2-ME (Gibco), and 100 U/ml penicillin/streptomycin. HTL cells were provided by G. Barnea (Brown University, Providence, RI) and maintained in DMEM supplemented with 10% FBS, 2 mM L-glutamine, 100 U/ml penicillin/streptomycin, and 1 µg/ml puromycin. In Tango assays for the association of COMMD8 with CXCR4 and its mutants, HTL cells were maintained in serum-free Pro293a medium (Lonza) supplemented with 2 mM L-glutamine and 1 µg/ml puromycin. The sex of HEK293 cells and their derivative HTL cells is female (Lin et al., 2014), whereas that of 2PK-3 cells has not been reported. Authentication of HEK293, 2PK-3, and HTL cells was performed by their sources. Authentication of Phoenix-E cells was performed by confirming that the cell line contained the necessary gag, pol, and env-encoding constructs. Mycoplasma contamination of HEK293, 2PK-3, and HTL cells was tested by their sources. Phoenix-E cells were not tested for mycoplasma contamination since the cell line itself was not being tested for functional properties.

### Yeast two-hybrid screening

Yeast two-hybrid screening was performed using the Matchmaker two-hybrid system 3 (Takara Bio). To construct a bait plasmid, the C-terminal tail of human CXCR4 (residues 306–352) was cloned into the pGBKT7 vector downstream of a Gal4 DNA-binding domain. Yeast strain AH109 was transformed with the bait plasmid and mated with yeast strain Y187 that was pretransformed with a human bone marrow cDNA library cloned in the pGADT7-Rec vector downstream of a Gal4-activating domain (Takara Bio). Positive clones were chosen from screening of 2 × 10^7^ clones, and pGADT7-Rec library plasmids were recovered from individual clones. The cDNA insert was sequenced and then characterized using a BLAST program (National Center for Biotechnology Information).

### Plasmid construction

The MSCV2.2 retroviral vector expressing mouse CXCR4, CCR7, or S1PR1 with an N-terminal Flag tag upstream of an internal ribosomal entry site and a cytoplasmic domain–truncated human CD4 as an expression reporter was provided by J.G. Cyster. cDNAs encoding mouse CXCR5, EBI2, β_2_AR, and C terminus (residues 313–359)–truncated CXCR4 (CXCR4ΔCt) with an N-terminal Flag tag and mouse CXCR4 with an N-terminal Myc tag were also cloned into this vector. The MSCV2.2 vector encoding GFP, truncated human CD4, or Thy-1.1 reporter downstream of an internal ribosomal entry site was used to express the following mouse proteins with a Myc or hemagglutinin (HA) tag fused to their C termini: COMMD8, COMMD3, COMMD8ΔC (residues 1–116), COMMD8ΔN (residues 116–183), COMMD3ΔC (residues 1–123), COMMD3ΔN (residues 123–195), GRK2, GRK5, and GRK6. For Tango assays, we constructed the pcDNA3 vector to express the following Flag-tagged mouse GPCRs and their mutants that were C-terminally fused via a TEV protease cleavage site to tTA: CXCR4, CXCR4 Δ310-327, CXCR4 Δ328-347, CXCR4 Δ348-359, CXCR4 S353-355A, β_2_AR, and β_2_AR Δ365-418. We also constructed the pcDNA vector encoding the Myc-tagged COMMD8 that was C-terminally fused to TEV protease. The integrity of all constructs was verified by sequencing.

### Retroviral transduction

Phoenix-E packaging cells were transfected with the MSCV2.2 constructs using Lipofectamine 2000 (Thermo Fisher Scientific), and supernatants containing the retrovirus were collected. 2PK-3 cells were spin-infected with retroviral supernatants and polybrene (4 µg/ml; Sigma-Aldrich) at 2,450 rpm in an AX-311 centrifuge (TOMY) for 2 h at 25°C as described previously (Reif et al., 2002).

### Gene deletion in cell lines

Sense (S) and antisense (AS) oligonucleotides encoding the following single guide RNAs were cloned into the pX330 vector (42230; Addgene) containing neomycin or puromycin resistance gene (coding sequences underlined): sg*COMMD8*_S, 5′-CAC​CAAG​AGG​GGA​CGC​CCT​TGT​GG-3′ and sg*COMMD8*_AS, 5′-AAA​CCCA​CAA​GGG​CGT​CCC​CTC​TT-3′; sg*COMMD3*_S, 5′-CAC​CGAC​TCT​GGA​ATG​CCG​CCC​GG-3′ and sg*COMMD3*_AS, 5′-AAA​CCCG​GGC​GGC​ATT​CCA​GAG​TC-3′; sg*Commd8*_S, 5′-CAC​CAAG​AGG​GGA​CGC​CGC​TTT​GG-3′ and sg*Commd8*_AS, 5′-AAA​CCCA​AAG​CGG​CGT​CCC​CTC​TT-3′; sg*Commd3*_S, 5′-CAC​CGCT​GGA​CGC​CCG​GGC​GGA​CG-3′ and sg*Commd3*_AS, 5′-AAA​CCGT​CCG​CCC​GGG​CGT​CCA​GC-3′; sg*Cxcr4*_S, 5′-CAC​CACT​CAC​ACT​GAT​CGG​TTC​CA-3′ and sg*Cxcr4*_AS, 5′- AAA​CTGG​AAC​CGA​TCA​GTG​TGA​GT-3′; sg*Grk6*_S, 5′-CAC​CAGC​GAA​CAC​GGT​GCT​ACT​CA-3′ and sg*Grk6*_AS, 5′-AAA​CTGA​GTA​GCA​CCG​TGT​TCG​CT-3′.

HEK293 and 2PK-3 cells were transfected with the pX330 construct using Lipofectamine 2000 and a pulse generator CUY21Vitro-EX (BEX), respectively, and selected with G418 (0.5–1 mg/ml; Nacalai Tesque) or puromycin (1–2.5 µg/ml; Gibco). Transfected HEK293 cells that survived the selection were expanded and validated by immunoblotting (IB) for target protein deletion. Transfected 2PK-3 cells that survived the selection were plated in 96-well plates using a limited dilution method with approximately one cell per well. Single cell clones were expanded and screened by IB for target protein depletion. Positive clones were further validated by confirming that the targeted exon contained out-of-frame mutations by genomic DNA sequencing. Cells transfected with the empty pX330 vector served as the control.

### Antibodies

Antibodies against Myc tag (9B11), K48-linked polyubiquitin (4289), Na, K-ATPase (3010), IκB-α (9242), β-arrestin-2 (C16D9, 3857), β-arrestin-1 (D8O3J, 12697), phospho-ERK1/2 (9101), and phospho-p38 (9211) were purchased from Cell Signaling Technology. Antibodies against COMMD8 (N-15), GAPDH (FL-335), GRK6 (C-20), and p38 (N-20) were from Santa Cruz Biotechnology. Antibodies against COMMD3 (HPA036584) and Flag tag (M2) were from Sigma-Aldrich. Antibodies against active (26901) and total (26003) Gα_i_ were from New East Bioscience. Antibodies against HA tag (3F10), ERK1/2 (216703), and CXCR4 (2B11, used for IP) were from Roche, R&D Systems, and BD Biosciences, respectively. Antibodies against mouse Ig isotypes were from SouthernBiotech. An anti-mouse IgM F(ab′)_2_ was from Jackson ImmunoResearch Laboratories. A rabbit polyclonal antibody recognizing GRK6-phosphorylated S355/356 of human and mouse β_2_AR was from Thermo Fisher Scientific. Rabbit polyclonal antibodies recognizing GRK6-phosphorylated S324/325 or S330 of human CXCR4 (corresponding to S331/332 or S337 of mouse CXCR4; Busillo et al., 2010) were provided by J.L. Benovic (Thomas Jefferson University, Philadelphia, PA). A rabbit polyclonal antibody recognizing GRK2-phosphorylated S346/347 of human CXCR4 (corresponding to S353/354 of mouse CXCR4) was generated as described previously (Mueller et al., 2013).

For flow cytometry and immunofluorescence, fluorophore- or biotin-labeled antibodies against the following molecules (BioLegend) were used: CD4 (GK1.5), CD19 (6D5), CD21/CD35 (7E9), CD23 (B3B4), CD44 (IM7), CD45.1 (A20), CD45.2 (104), CD93 (AA4.1), CD117 (2B8), CD138 (281–2), CCR7 (4B12), IgD (11-26c.2a), IgD^a^ (AMS-9.1), IgM (RMM-1), GL7, and MAdCAM-1 (MECA-367). Fluorophore- or biotin-labeled antibodies against CD95 (Jo2), CXCR4 (2B11), CXCR5 (2G8), and IgD^b^ (217–170) were purchased from BD Biosciences. The Alexa Fluor 647–conjugated anti-Myc tag antibody (9B11) and FITC–conjugated anti-Flag tag antibody (M2) were purchased from Cell Signaling Technology and Sigma-Aldrich, respectively. The goat polyclonal antibody against EBI2 (A-20) was from Santa Cruz Biotechnology.

### IB analysis

To examine the subcellular distribution of COMMD8 and COMMD3, mouse spleen cells were washed three times and then incubated for 4 h at 37°C in HBSS (Wako) containing 10 mM Hepes (Dojindo). After stimulation with CXCL12 (300 ng/ml; R&D Systems), cells were homogenized in detergent-free homogenization buffer (250 mM sucrose, 1 mM EDTA, 10 mM 2-ME, and 10 mM Tris-HCl, pH 8.0) supplemented with a protease inhibitor cocktail (Nacalai Tesque), and the cytosolic fraction was separated by centrifugation at 120,000 ×*g* for 1 h. The pellet was lysed with 1% Triton X-100 (Wako) in homogenization buffer and then centrifuged at 20,000 ×*g* for 15 min to obtain the membrane-rich fraction. To detect endogenous COMMD8 and COMMD3 in B cells, Fo B cells were isolated from mouse spleens by cell sorting and lysed with 1% NP-40 lysis buffer (1% NP-40 from Nacalai Tesque, 150 mM NaCl, and 10 mM Tris-HCl, pH 7.2) containing the protease inhibitor cocktail. To assess degradation of COMMD8 and COMMD3, control and COMMD3- or COMMD8-deficient 2PK-3 cells that were transduced with Myc-tagged COMMD8 or COMMD3 were cultured with 10 µg/ml cycloheximide (Nacalai Tesque) in the presence or absence of 10 µM MG132 (Merck Millipore). To assess IκB degradation or MAPK activation, B cells were isolated from mouse spleens by negative selection with CD43 microbeads (Miltenyi Biotec) on an AutoMACS Pro Separator (Miltenyi Biotec) and incubated for 2 h at 37°C in HBSS containing 10 mM Hepes. After stimulation with the anti-mouse IgM F(ab′)_2_ (10 µg/ml) or CXCL12 (30 ng/ml), the cells were lysed with Mg^2+^ Lysis Buffer (Merck Millipore) containing protease and phosphatase inhibitor cocktails (Nacalai Tesque). To assess β_2_AR-mediated MAPK activation, 2PK-3 cells transduced with Flag-tagged β_2_AR were stimulated with isoproterenol (1 µM; Sigma-Aldrich) after incubation for 3 h at 37°C in HBSS containing 10 mM Hepes and then lysed with the Mg^2+^ Lysis Buffer.

Reduced samples were resolved in NuPAGE Bis-Tris gels (Thermo Fisher Scientific) and transferred to Immobilon-P membranes (Merck Millipore). The membranes were blocked with 2.5% dry skim milk in Tris-buffered saline (TBS) containing 0.1% Tween 20 and blotted with primary antibodies followed by HRP–conjugated secondary antibodies (Jackson ImmunoResearch Laboratories). To detect phosphorylation of MAPKs, membranes were blocked with 1% globulin-free BSA (Wako) in TBS containing 0.1% Tween 20. Images were acquired with a luminescence image analyzer (Amersham Imager 600; GE Healthcare). As a measure of protein levels, band density was analyzed using ImageJ software (National Institutes of Health). Except for Fig. 2 A, protein levels were determined as the ratio to the levels of untreated samples and normalized to the levels of loading controls.

### IP assay

To assess the association between COMMD8 and COMMD3, mouse spleen cells or transduced 2PK-3 cells were washed with PBS and then lysed with digitonin lysis buffer (1% digitonin from Sigma-Aldrich, 0.125% NP-40, 150 mM NaCl, and 10 mM Tris-HCl, pH 7.2) containing the protease inhibitor cocktail. To assess ubiquitination of COMMD8 and COMMD3, cells were washed with PBS and then lysed with 0.5% NP-40 lysis buffer (0.5% NP-40, 150 mM NaCl, and 10 mM Tris-HCl, pH 7.2) containing the protease inhibitor cocktail and 10 mM N-ethylmaleimide (Sigma-Aldrich).

To assess the interaction of COMMD8 or COMMD3 with GPCRs or GRKs, cells were washed five times and then incubated for 30 min at 37°C in Roswell Park Memorial Institute (RPMI) medium (Wako) containing 0.5% fatty acid–free BSA (Merck Millipore). Transduced 2PK-3 cells were stimulated with CXCL12 (100 ng/ml), CXCL13 (300 ng/ml; R&D Systems), CCL19 (100 ng/ml; R&D Systems), 7α,25-HC (30 nM; Sigma-Aldrich), S1P (100 nM; Sigma-Aldrich), or clenbuterol (100 µM; Sigma-Aldrich). B cells were isolated from mouse spleens by negative selection using the AutoMACS Pro Separator and were stimulated with CXCL12 (600 ng/ml). To assess the recruitment of β-arrestin-2 to CXCR4 or β_2_AR, cells were washed three times and then incubated for 30 min or 2 h at 37°C in HBSS containing 10 mM Hepes, followed by stimulation with CXCL12 (100 ng/ml) or isoproterenol (1 µM). To assess site-specific phosphorylation of CXCR4 or β_2_AR, cells were incubated for 1–3 h at 37°C in HBSS containing 10 mM Hepes and then stimulated with CXCL12 (2PK-3 cells, 100 ng/ml; B cells, 300 ng/ml) or isoproterenol (10 µM). To assess the recruitment of GRK6 to CXCR4, cells were stimulated with CXCL12 (100 ng/ml) after incubation for 13 h at 37°C in DMEM containing 0.01% fatty acid–free BSA. After stimulation of GPCRs, cells were lysed with the digitonin lysis buffer containing protease and phosphatase inhibitor cocktails. To assess Gα_i_ activation, cells were stimulated with CXCL12 (600 ng/ml) after incubation for 5 h at 37°C in HBSS containing 10 mM Hepes and then lysed with Triton X-100 lysis buffer (1% Triton X-100, 150 mM NaCl, 1 mM EDTA, and 50 mM Tris-HCl, pH 7.4) containing the protease inhibitor cocktail.

Cell lysates were precleared for 30 min at 4°C with protein A or G sepharose (GE Healthcare), followed by incubation for 3–5 h at 4°C with anti-Flag M2 beads (Sigma-Aldrich) or antibodies plus protein A or G sepharose. Beads were washed with lysis buffer three to five times. Samples were eluted by incubation with Flag peptides (Sigma-Aldrich) for 30 min, Myc peptides (Sigma-Aldrich) for 1 h on ice, or NuPAGE LDS Sample Buffer (Thermo Fisher Scientific) for 30 min at room temperature or 10 min at 70°C. Reduced samples were analyzed by IB as described in the method for IB analysis.

### Measurement of cAMP

To assess CXCR4-mediated inhibition of cAMP production, control and COMMD8- or COMMD3-deficient HEK293 cells were transfected with plasmids encoding Flag-tagged CXCR4 and a luciferase-based cAMP biosensor, GloSensor (pGloSensor-22F; Promega) at a 1:1 ratio. 24 h after transfection, cells were collected in DMEM containing 1% FBS and seeded at a density of 8 × 10^4^ cells per well in 96-well clear-bottom white plates (3610; Corning). After 24 h of culture, the medium was replaced with 100 µl HBSS containing 0.1% fatty acid–free BSA and 10 mM Hepes plus 2% GloSensor cAMP reagent (Promega), and the cells were incubated for 2 h in the dark at room temperature. 5 min after stimulation with CXCL12, cells were treated with forskolin (10 µM; Sigma-Aldrich) for 15 min. To measure cAMP production mediated by endogenous β_2_AR, control and mutant HEK293 cells were transfected with pGloSensor-22F in 96-well clear-bottom white plates. After 24 h, the cells were equilibrated with 2% GloSensor cAMP reagent for 2 h as described above, and then stimulated with isoproterenol for 1 min. GloSensor luciferase activities were measured in triplicate with a GloMax microplate reader (Promega) in luminescence mode.

### Flow cytometry and cell sorting

Lymphoid tissues were disrupted by passing through a 40-µm cell strainer (BD Biosciences). For blood samples, red blood cells were lysed with ammonium chloride potassium buffer. Single cells were stained with fluorophore- or biotin-labeled antibodies. Data were acquired on a FACSVerse cytometer (BD Biosciences) and analyzed with FlowJo software (Tree Star) to detect the following populations: pro-B cells (CD19^+^IgM^−^CD117^+^), preB cells (CD19^+^IgM^−^CD117^−^), immature B cells (CD19^+^IgM^+^IgD^−^ or IgD^lo^), and mature B cells (CD19^+^IgM^+^IgD^hi^) in the bone marrow, blood, and LNs of unimmunized mice; T1 B cells (CD19^+^CD93^+^CD21^lo^CD23^lo^), T2 B cells (CD19^+^CD93^+^CD21^hi^CD23^hi^), Fo B cells (CD19^+^CD93^−^CD21^int^CD23^hi^), and marginal zone B cells (CD19^+^CD93^−^CD21^hi^CD23^lo^) in the spleen of unimmunized mice; and GC B cells (CD19^+^CD95^+^GL7^+^) and plasmablasts (CD44^hi^CD138^+^) in the spleen of immunized mice. NP-specific GC B cells were labeled with NP_23_-PE (Biosearch Technologies). To detect NP-specific plasmablasts, single cell suspensions were prepared from the spleen by digestion with collagenase type IV (Worthington Biochemical) as described previously (Yi et al., 2012). After labeling CD44 and CD138 on the cell surface, the cells were fixed and permeabilized with Cytofix/Cytoperm solution (BD Biosciences) and then labeled with NP_23_-PE. Surface expression of chemokine receptors on Fo B cells was analyzed after incubation in RPMI medium containing 0.5% fatty acid–free BSA for 30 min at 37°C to resensitize the receptors and reproduce the cellular conditions for the transwell migration assay. Cell sorting was performed on a FACSAria II (BD Biosciences) to isolate the following populations: pro-B cells (CD19^+^IgM^−^CD117^+^), preB cells (CD19^+^IgM^−^CD117^−^), immature B cells (CD19^+^IgM^+^IgD^−^ or IgD^lo^), and mature B cells (CD19^+^IgM^+^IgD^hi^) in the bone marrow; and T1 B cells (CD19^+^CD93^+^CD21^lo^CD23^lo^), T2 B cells (CD19^+^CD93^+^CD21^hi^CD23^hi^), Fo B cells (CD19^+^CD93^−^CD21^int^CD23^hi^), and marginal zone B cells (CD19^+^CD93^−^CD21^hi^CD23^lo^) in the spleen.

### Immunofluorescence microscopy

To visualize the subcellular localization of COMMD8 and COMMD3, HEK293 cells were transfected with plasmids encoding Myc-tagged COMMD8 or COMMD3 and Flag-tagged CXCR4 or β_2_AR. 24 h after transfection, the cells were seeded on collagen-coated coverslips (Iwaki). After 12–20 h, the cells were incubated in DMEM for 2–4 h at 37°C and then stimulated with CXCL12 (300 ng/ml) or isoproterenol (10 µM) for 1 min. The stimulated cells were fixed with 4% paraformaldehyde in PBS with Ca^2+^ and Mg^2+^ and permeabilized in PBS containing 1% BSA and 0.005% Triton X-100. The cells were stained with the Alexa Fluor 647–conjugated anti-Myc tag antibody, FITC–conjugated anti-Flag tag antibody, and DAPI. To assess follicular localization of transferred B cells, B cells were isolated from the mouse spleen by negative selection using the AutoMACS Pro Separator and labeled with 2 µM CFSE (Thermo Fisher Scientific) in DMEM containing 1% FBS for 10 min at 37°C. Each recipient mouse received 10^7^ cells, and the spleen was harvested at 24 h after the cell transfer. Unfixed fresh frozen sections of 10 µm in thickness were blocked with Protein Block Serum-Free (Dako) and stained to detect IgD, CD4, IgD^a^, IgD^b^, MAdCAM-1, and GL7. Cells or sections were mounted with FluorSave Reagent (Merck Millipore). Images were acquired at room temperature with Fluoview FV10-ASW version 3.00 using a Fluoview FV1000 inverted confocal microscope (Olympus) equipped with plan apochromat UPLSAPO objectives (10× with 0.40 numerical aperture [NA], 20× with 0.75 NA, or 100× oil immersion with 1.40 NA). Colocalization of fluorescent signals was analyzed with the plug-in “RG2B Colocalization” for ImageJ software. Localization of CFSE signals in the B cell follicles versus red pulp was analyzed with ImageJ software and normalized to the area ratio of the two regions.

### Quantitative PCR analysis

Total RNA was extracted from sorted cells with a Nucleospin RNA kit (Macherey-Nagel) and reverse-transcribed with ReverTra Ace qPCR RT master mix (Toyobo). Quantitative PCR was performed with SYBR Premix Ex Taq II on a Thermal Cycler Dice Real Time System II (Takara Bio). The following forward (F) and reverse (R) primers were used: Commd8_F, 5′-GGA​ATT​CAG​CTG​AGT​GGA​AGC​A-3′ and Commd8_R, 5′-TTC​TTG​GTG​ACA​TGA​ATT​CAG​TT-3′; Commd3_F, 5′-AAA​TTT​GAC​CGA​GAG​CGA​ATA​GAA​C-3′ and Commd3_R, 5′-CTG​GTA​CTC​CAA​GCG​CCA​AG-3′; Gapdh_F, 5′-TGT​GTC​CGT​CGT​GGA​TCT​GA-3′ and Gapdh_R, 5′-TTG​CTG​TTG​AAG​TCG​CAG​GAG-3′.

### Transwell migration assay

Mouse spleen cells were treated with ammonium chloride potassium buffer to lyse red blood cells, washed five times, and then incubated for 30 min at 37°C in RPMI medium containing 0.5% fatty acid–free BSA. Cells were seeded in the upper chambers of transwells (3421; Corning) and tested for transmigration across filters with 5-µm pores for 3 h at 37°C in response to the following chemoattractants in the lower chambers: CXCL12, CXCL13, CCL19, 7α,25-HC, or S1P (Sigma-Aldrich). To assess the role of MAPKs in B cell chemotaxis, cells were pretreated with ERK inhibitor U0126 (Merck Millipore) or p38 inhibitor SB203580 (Sigma-Aldrich) for 10 min and seeded in the transwells with the lower chambers containing CXCL12 (300 ng/ml) and the same concentrations of the inhibitor used for pretreatment. B cells that migrated to the lower chambers were enumerated by flow cytometry, and chemotactic responses were determined as percentages of their numbers relative to the numbers of input cells.

### Receptor internalization

Mouse spleen cells were prepared as described for the transwell migration assay. Cells were incubated with a chemoattractant for 60 min at 37°C in RPMI medium containing 0.5% fatty acid–free BSA and then briefly exposed to 0.05 M glycine-HCl buffer (pH 3.0) containing 0.1 M NaCl to remove the chemoattractant from the receptors. After washing, surface levels of the receptors on Fo B cells were analyzed by flow cytometry. Internalization of the receptors was determined by the reduction of their surface expression.

### Intracellular calcium response

To measure BCR-induced intracellular calcium responses, mouse LN cells were loaded with 0.5 µM Cal-520 AM (AAT Bioquest) and 5 µM Fura Red AM (Invitrogen) plus 0.1% Pluronic F-127 (Invitrogen) in DMEM containing 1% FBS for 45 min at 37°C and then labeled with the allophycocyanin–conjugated anti-CD19 antibody on ice. The cells were warmed at 25°C for 10 min before stimulation with the anti-mouse IgM F(ab′)_2_ (10 µg/ml). To measure intracellular calcium responses to CXCL12 (200 ng/ml), we used DMEM containing 0.5% fatty acid–free BSA instead of the FBS-containing medium. Changes of fluorescence intensity in CD19^+^ cells were monitored on a FACSCant II cytometer (BD Biosciences).

### Tango assay

Tango assays were performed as described previously (Barnea et al., 2008). HTL cells were transfected with the GPCR-TEV cleavage site-tTA plasmid, COMMD8-TEV protease plasmid, and pGL4.74 (hRluc/TK) Renilla luciferase expression vector (Promega) at a 10:5:1 ratio. 1 d after transfection, cells were collected in serum-free DMEM and seeded at a density of 6.4 × 10^4^ cells per well in 96-well clear-bottom white plates. After 2–6 h of culture, cells were stimulated with CXCL12 (10 ng/ml) or isoproterenol (1 nM). Paroxetine (Merck Millipore) treatment was started 1 h before ligand stimulation. After 12 h, tTA-induced firefly luciferase activity was determined using the Dual-Glo luciferase assay system (Promega) and normalized to Renilla luciferase activity. Luminescence was measured in triplicate with the GloMax microplate reader in luminescence mode.

### Lymphocyte entry to LNs

Cells were collected from cervical, axillary, brachial, inguinal, and mesenteric LNs of CD45.2 control, *Commd8*Δ, *Commd3*Δ, and CD45.1 WT mice. CD45.2 and CD45.1 cells were mixed at a 1:1 ratio, labeled with 0.1 µM CFSE in DMEM containing 1% FBS for 15 min at 37°C, and then transferred to CD45.2 WT mice (3 × 10^7^ cells per mouse). Peripheral (inguinal, axillary, and brachial) LNs were collected from the recipients at 90 min after the cell transfer. Cells were subjected to flow-cytometric analysis of CFSE-labeled B cells (CD19^+^) and CD4^+^ T cells (CD4^+^). The ratio of CD45.2 to CD45.1 cells was normalized to that of input populations.

### Lymphocyte egress from LNs

Cells were collected from cervical, axillary, brachial, inguinal, and mesenteric LNs of CD45.2 control, *Commd8*Δ, *Commd3*Δ, and CD45.1 WT mice. CD45.2 and CD45.1 cells were mixed at a 1:1 ratio, labeled with 0.5 µM CFSE in DMEM containing 1% FBS for 15 min at 37°C, and then transferred to CD45.2 WT mice (3 × 10^7^ cells per mouse). At 20–24 h after the cell transfer, lymphocyte entry to LNs was blocked by i.v. injection of neutralizing antibodies against α4 (PS/2) and αL (M17/4) integrins (both purchased from Bio X Cell) at 100 µg each per mouse (Lo et al., 2005). CFSE-labeled B cells (CD19^+^) and CD4^+^ T cells (CD4^+^) remaining in peripheral LNs were enumerated by flow cytometry at 12 h after the entry blockade. The ratio of CD45.1 to CD45.2 cells that remained in the LNs at 12 h after entry blockade was normalized to the ratio of the cells that had resided in the LNs at the time of entry blockade.

### Immunization

Mice received an i.p. injection of 100 µg NP_31_-CGG (Biosearch Technologies) in Imject Alum (Thermo Fisher Scientific) on day 0 and 50 µg NP_31_-CGG in PBS on day 28. Sera were collected every week after immunization to measure anti-NP antibody titers by ELISA. The spleen was collected at 7 and 10 d after immunization and subjected to flow-cytometric analysis of GC B cells and plasmablasts. To assess positioning of antigen-activated B cells, control and *Commd8*Δ B1-8^hi^ mice received an i.v. injection of 30 µg NP_23_-PE. Spleens were harvested at the indicated times after immunization, and the sections were analyzed by immunofluorescence microscopy. NP-binding B cells were identified by PE fluorescence.

### ELISA

Maxisorp immunoplates (Thermo Fisher Scientific) were coated with NP_50_-BSA (Biosearch Technologies) or antibodies against mouse Ig isotypes, followed by blocking with 0.5% BSA in PBS containing 0.05% Tween-20. Serially diluted sera were added to the wells, followed by incubation for 5–6 h at room temperature. After washing with PBS containing 0.05% Tween-20, HRP–conjugated antibodies against mouse Ig isotypes (SouthernBiotech) were added to the wells, followed by overnight incubation at 4°C. The HRP activity was detected with tetramethylbenzidine substrate (Kirkegaard & Perry Laboratories), and absorbance at 450 nm was measured using an iMark microplate reader (Bio-Rad) after quenching the reaction with 1 N HCl.

### Statistical analysis

GraphPad Prism 6 (GraphPad Software) was used for all statistical analyses. Statistical parameters, including the exact value of *n* with the description of what *n* represents, the mean, and dispersion (SD or SEM), are reported in the figures and figure legends. Statistical significance was determined by two-tailed paired or unpaired Student’s *t* test for two groups or one-way ANOVA with Tukey’s post hoc test for multiple groups. A P value of <0.05 was considered to be statistically significant. In the measurement of cAMP levels, data were plotted and fit using nonlinear regression.

### Online supplemental material

Fig. S1 provides supplementary information for the characterization of COMMD8 and COMMD3. Fig. S2 provides supplementary information for the role of the COMMD3/8 complex in B cell migration and humoral immune responses. Fig. S3 shows B cell populations and serum antibody titers in *Commd8*Δ and *Commd3*Δ mice. Fig. S4 shows CXCR4 signaling in COMMD3- and β-arrestin–deficient B cells. Fig. S5 provides supplementary information for the role of the COMMD3/8 complex in GRK6-mediated phosphorylation of CXCR4.

## Supplementary Material

Supplemental Materials (PDF)
