# Supplementary material for: The COMMD3/8 complex determines GRK6 specificity for chemoattractant receptors
Source: J Exp Med. 2019 May 14;216(7):1630–47. doi: 10.1084/jem.20181494 (PMC6605747; doi:10.1084/jem.20181494)
Supplement: Supplemental Materials (PDF) [file JEM_20181494_sm.pdf]

JEM

Supplemental material

Nakai et al., https://doi.org/10.1084/jem.20181494

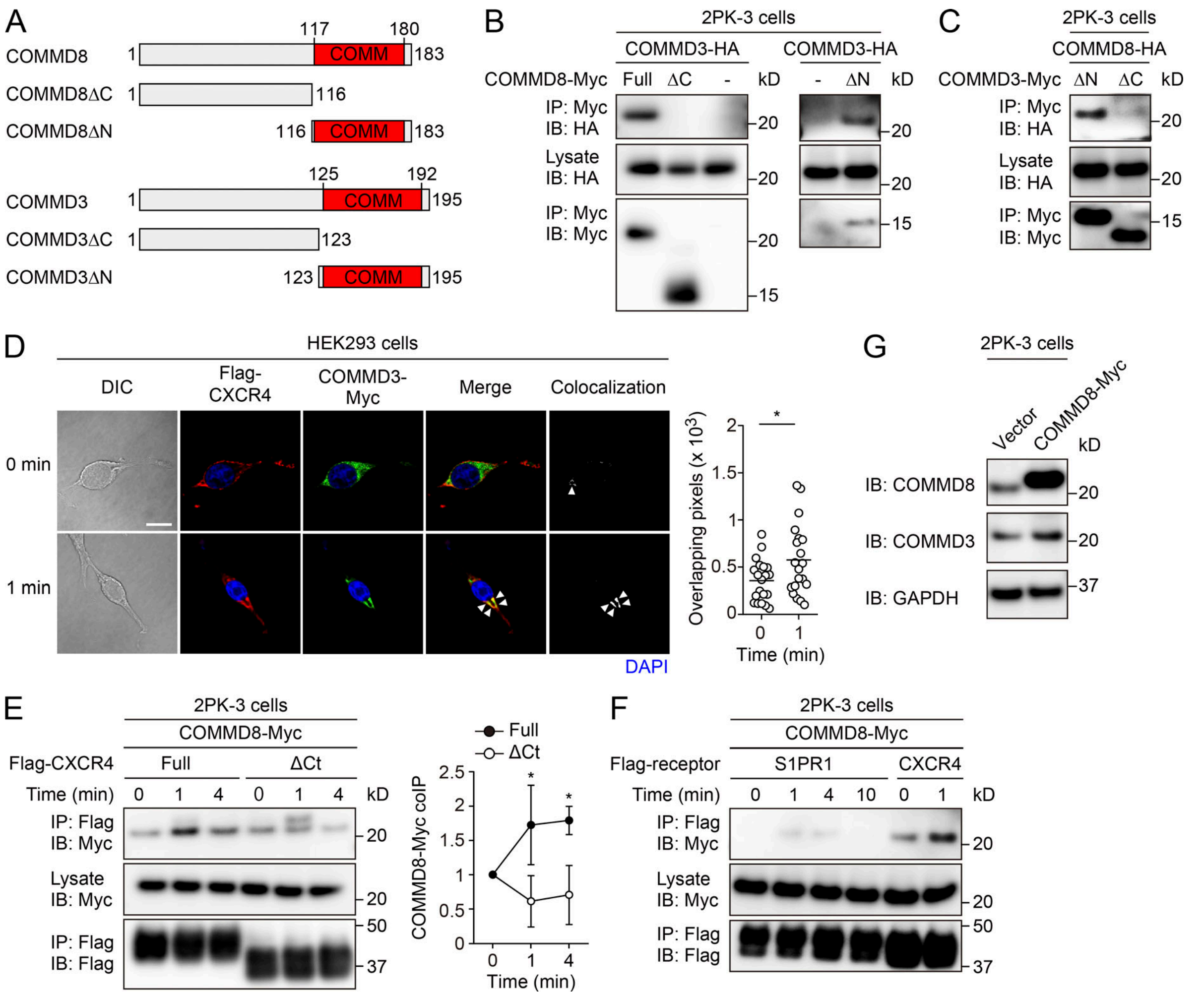

Figure S1. **Characterization of COMMD8 and COMMD3. (A)** Diagrams of full-length mouse COMMD8, COMMD3, and their C-terminal (ΔC) or N-terminal (ΔN) truncation mutants used in B and C. **(B and C)** IP assays for the interaction of full-length COMMD3 (B) or COMMD8 (C) with ΔC and ΔN mutants of COMMD8 (B) or COMMD3 (C) in 2PK-3 cells. **(D)** Confocal microscopy for the subcellular localization and colocalization of Flag-tagged CXCR4 (red) and Myc-tagged COMMD3 (green) in HEK293 cells before and at 1 min after CXCL12 treatment. Colocalization of the signals (arrowheads) was quantified as in Fig. 1 C. Each symbol represents an individual cell, and bars indicate means (0 min, *n* = 20; 1 min, *n* = 20). Representative images are shown. Bar, 10 µm. **(E)** IP assay for the interaction of Myc-tagged COMMD8 with a C terminus–truncated mutant of Flag-tagged CXCR4 (ΔCt) in 2PK-3 cells stimulated with CXCL12. Flag-tagged full-length CXCR4 (Full) served as a positive control. **(F)** IP assay for the interaction of Myc-tagged COMMD8 with Flag-tagged S1PR1 in 2PK-3 cells stimulated with S1P. Flag-tagged CXCR4 served as a positive control. **(G)** IB analysis for the levels of COMMD8 and COMMD3 proteins in 2PK-3 cells transfected with vector or Myc-tagged COMMD8. Data are representative of three (B, C, and F) or two (G) independent experiments or pooled from three independent experiments (D). Error bars represent the mean ± SD of three independent experiments, and representative blots are shown (E). *, $P < 0.05$. The P values were obtained by two-tailed unpaired *t* test (D and E). coIP, coimmunoprecipitation; DIC, differential interference contrast.

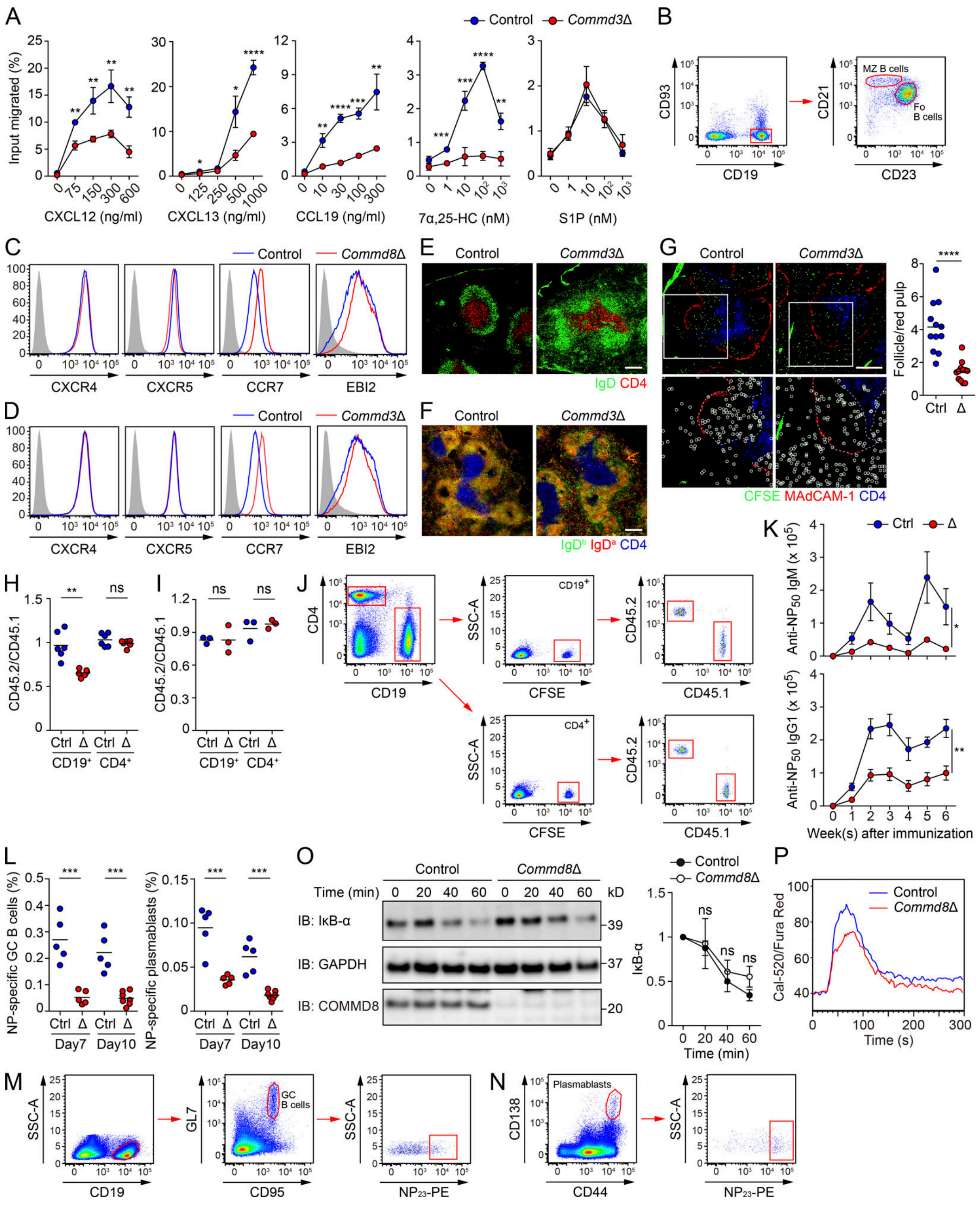

Figure S2. **COMMD3 deficiency impairs B cell migration and humoral immune responses.** **(A)** Chemotactic responses of control and *Commd3*Δ B cells were assessed by transwell migration of Fo B cells toward CXCL12, CXCL13, CCL19, 7α,25-HC, and S1P. Data are shown as the mean ± SD of triplicates. **(B)** Gating strategy for splenic B cells in Fig. 3 A; Fig. S2, A, C, and D; and Fig. S4, I–M. MZ, marginal zone. **(C and D)** Flow-cytometric analysis for the surface expression of chemoattractant receptors on control and *Commd8*Δ (C) or *Commd3*Δ (D) Fo B cells. **(E)** Spleen tissues from control and *Commd3*Δ mice were analyzed as in Fig. 3 B. **(F)** Distribution of control and *Commd3*Δ B cells in the spleen of mixed bone marrow chimeras (20% Igh^b control or *Commd3*Δ plus 80% Igh^a WT) was analyzed as in Fig. 3 C. **(G)** Follicular localization of CFSE$^+$ control (Ctrl) and *Commd3*Δ (Δ) B cells was assessed as in Fig. 3 D. Each symbol represents an individual analyzed field, and bars indicate means (n = 12). **(H and I)** A mixture of CFSE-labeled LN cells (50% CD45.2 control or *Commd3*Δ plus 50% CD45.1 WT) was transferred into WT mice. LN entry (H) and egress (I) of CD45.2 cells relative to CD45.1 B cells (CD19$^+$) were assessed as in Fig. 3, E and F, respectively. **(J)** Gating strategy for CFSE-labeled, transferred lymphocytes in Fig. 3, E and F; and Fig. S2, H and I. **(K and L)** Serum antibody titers (K, shown as the mean ± SEM of seven control or six *Commd3*Δ mice) and the generation of GC B cells and plasmablasts (L, shown as the percentages among B cells and total lymphocytes, respectively) were measured at the indicated times after NP-CGG immunization of control and *Commd3*Δ mice. **(M and N)** Gating strategies for GC B cells (M) and plasmablasts (N) in Fig. 3 I and Fig. S2 L. **(O)** IB analysis for IκB degradation in control and *Commd8*Δ B cells after stimulation with an anti-IgM F(ab′)$_2$. Error bars represent the mean ± SD of three independent experiments, and representative blots are shown. **(P)** Intracellular calcium responses in control and *Commd8*Δ B cells after stimulation with an anti-IgM F(ab′)$_2$. Responses are plotted as the ratio of Cal-520 to Fura Red fluorescence. Littermate *Commd3*$^{f/+}$*Mb1*$^{Cre/+}$ (F–H), *Commd3*$^{f/f}$*Mb1*$^{+/+}$ (A, D, E, I, K, and L), or *Commd8*$^{f/f}$*Mb1*$^{+/+}$ (C, O, and P) mice served as the control. Data are representative of three (A) or two (C–F, I, and P) experiments, or pooled from two (G, H, K, and L) experiments. Each symbol represents an individual mouse, and bars indicate means (H, I, and L). *, P < 0.05; **, P < 0.01; ***, P < 0.001; ****, P < 0.0001; ns, not significant. The P values were obtained by two-tailed unpaired (A, G–I, L, and O) or paired (K) t test. Bars, 200 µm. SSC-A, side scatter area.

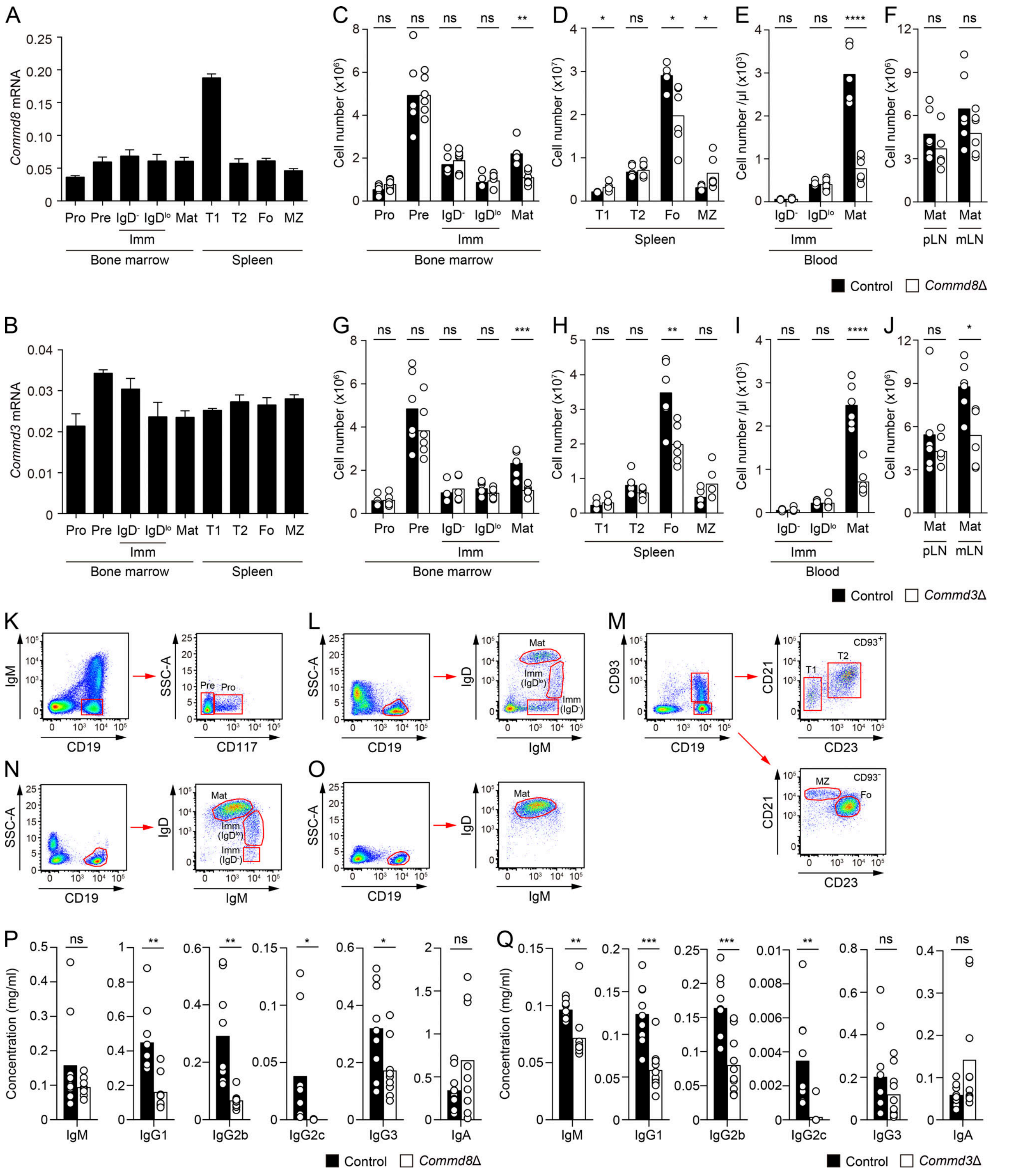

Figure S3. **Phenotypic analysis of *Commd8Δ* and *Commd3Δ* mice. (A and B)** Quantitative PCR analysis for the levels of *Commd8* (A) and *Commd3* (B) mRNAs relative to *Gapdh* expression in B cell populations in the bone marrow and spleen. Data are shown as the mean + SD of triplicates and representative of two independent experiments. **(C–J)** Flow-cytometric analysis for the abundance of B cell populations in the bone marrow (C and G), spleen (D and H), blood (E and I), and LNs (F and J) of *Commd8Δ* (C–F) or *Commd3Δ* (G–J) mice and littermate controls (*Commd8*$^{f/+}$*Mb1*$^{Cre/+}$ or *Commd3*$^{f/+}$*Mb1*$^{Cre/+}$). Data are pooled from five (C–F) or six (G–J) experiments and shown as the mean with symbols representing individual mice. **(K–O)** Gating strategies for B cell populations in the bone marrow (K and L), spleen (M), blood (N), and LNs (O). **(P and Q)** Serum concentrations of Ig isotypes in unimmunized *Commd8Δ* or *Commd3Δ* mice and littermate controls (*Commd8*$^{f/+}$*Mb1*$^{Cre/+}$ and *Commd8*$^{f/f}$*Mb1*$^{+/+}$, or *Commd3*$^{f/+}$*Mb1*$^{Cre/+}$ and *Commd3*$^{f/f}$*Mb1*$^{+/+}$). Data are pooled from two experiments and shown as the mean with symbols representing individual mice. *, P < 0.05; **, P < 0.01; ***, P < 0.001; ****, P < 0.0001; ns, not significant. The P values were obtained by two-tailed unpaired *t* test. Imm, immature; Mat, mature; T1, transitional type 1; T2, transitional type 2; MZ marginal zone, pLN, peripheral LN; mLN, mesenteric LN. SSC-A, side scatter area.

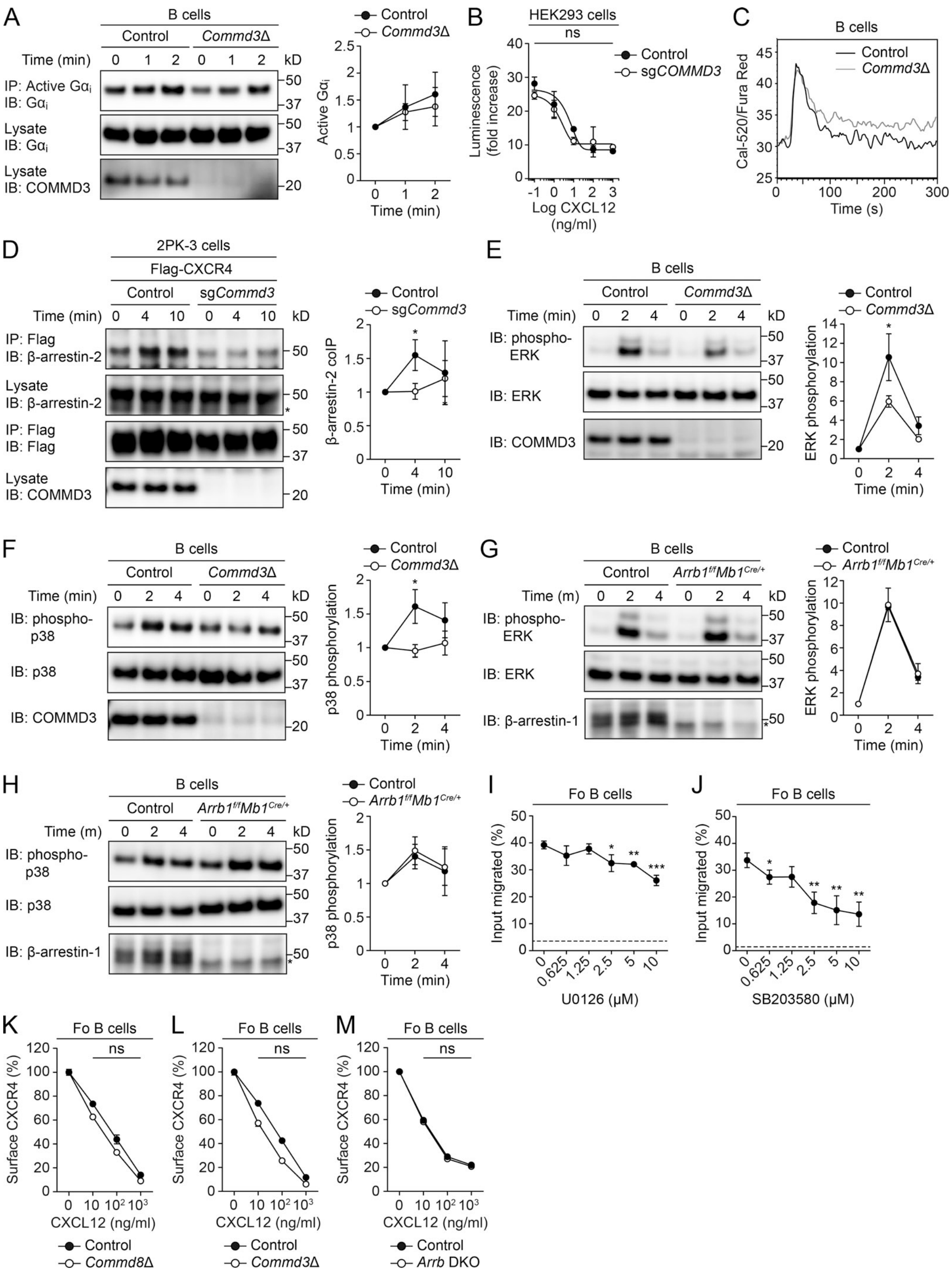

Figure S4. **CXCR4 signaling in COMMD3- and β-arrestin–deficient B cells. (A)** IB analysis for $G\alpha_i$ activation induced by CXCL12 in control and *Commd3Δ* B cells. **(B)** CXCR4-mediated inhibition of cAMP production was assessed by GloSensor in vector-transfected control and COMMD3-deficient (sg*COMMD3*) HEK293 cells expressing Flag-tagged CXCR4. Data are shown as the mean ± SD of triplicates and representative of three independent experiments. **(C)** Intracellular calcium responses to CXCL12 in control and *Commd3Δ* B cells. Data are representative of two independent experiments. **(D)** IP assay for the recruitment of endogenous β-arrestin-2 to Flag-tagged CXCR4 in vector-transfected control and COMMD3-deficient (sg*Commd3*) 2PK-3 cells after stimulation with CXCL12. **(E–H)** IB analysis for the phosphorylation of ERK (E and G) and p38 (F and H) in *Commd3Δ* (E and F) and β-arrestin-1–deficient ($Arrb1^{f/f}Mb1^{Cre/+}$; G and H) B cells after stimulation with CXCL12. **(I and J)** Transwell migration of Fo B cells toward CXCL12 in the presence of an inhibitor for ERK (U0126; I) or p38 (SB203580; J). Dashed lines indicate the levels of migration in the absence of CXCL12. **(K and L)** Flow-cytometric analysis for the ligand-induced internalization of CXCR4 in control and *Commd8Δ* (K) or *Commd3Δ* (L) Fo B cells. Surface levels of the receptors are shown as percentages relative to the initial levels. **(M)** Flow-cytometric analysis for the ligand-induced internalization of CXCR4 in Fo B cells doubly deficient of β-arrestin-1 and β-arrestin-2 ($Arrb1^{f/f}Arrb2^{-/-}Mb1^{Cre/+}$, *Arrb* DKO). B cells from the following littermate mice served as the control: $Commd3^{f/+}Mb1^{Cre/+}$ and $Commd3^{f/f}Mb1^{+/+}$ mice (A, C, E, F, and L), $Arrb1^{f/f}Mb1^{+/+}$ mice (G and H), $Commd8^{f/f}Mb1^{+/+}$ mice (K), or $Arrb1^{f/f}Arrb2^{+/+}Mb1^{+/+}$ mice (M). Error bars represent the mean ± SD of three (A and D–H) independent experiments, and representative blots are shown. Values represent the mean ± SD of triplicates and are representative of two independent experiments (I–M). *, $P < 0.05$; **, $P < 0.01$; ***, $P < 0.001$; ns, not significant. The P values were obtained by two-tailed unpaired (A and D–J) or paired (B and K–M) *t* test. Asterisks indicate nonspecific bands (D, G, and H). coIP, coimmunoprecipitation.

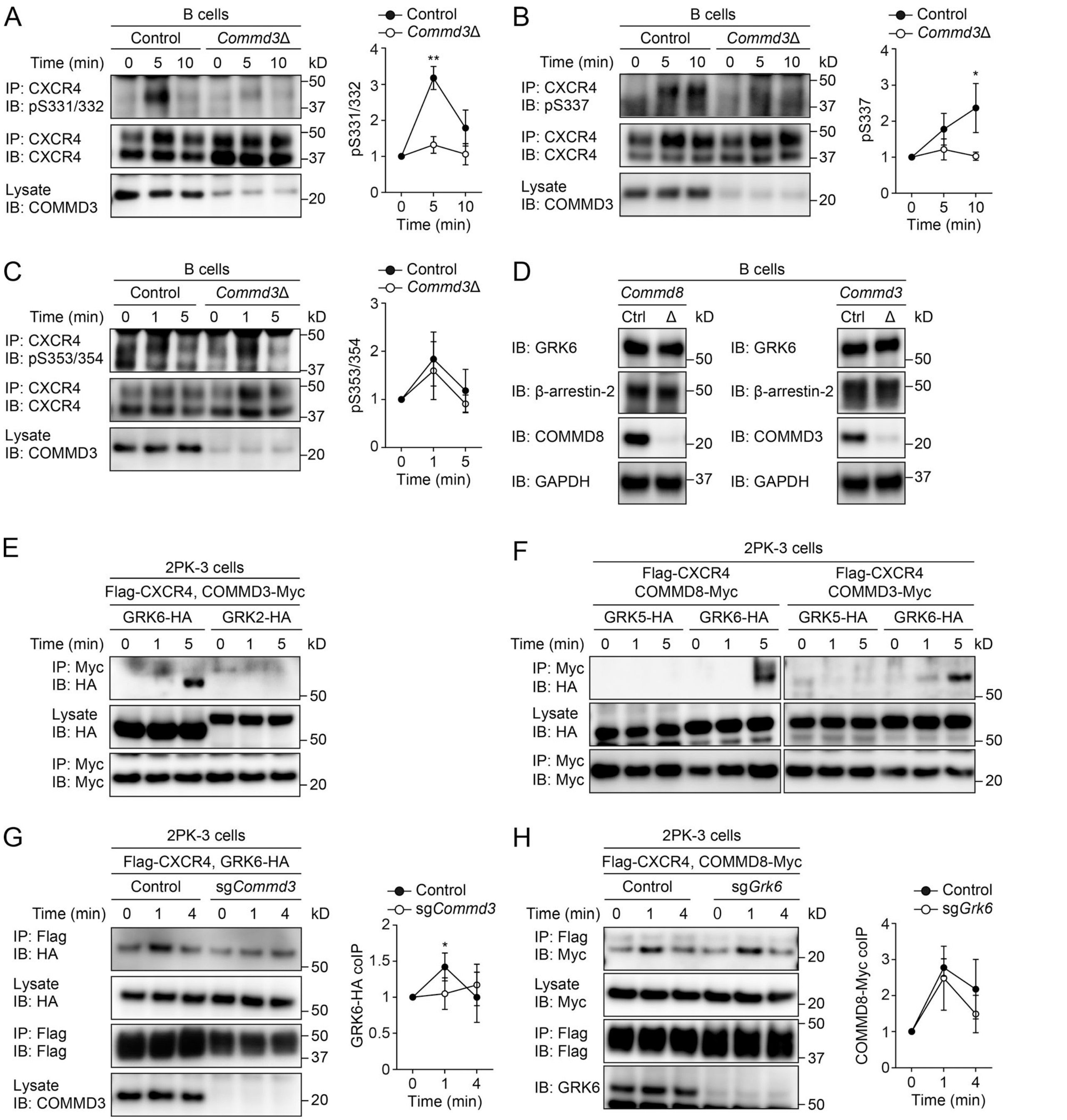

Figure S5. **COMMD3 is required for GRK6-mediated phosphorylation of CXCR4. (A–C)** IB analysis for the phosphorylation (p) at S331/332 (A), S337 (B), and S353/354 (C) of CXCR4 in control ($Commd3^{f/f}Mb1^{+/+}$) and *Commd3*Δ B cells after stimulation with CXCL12. **(D)** IB analysis for the levels of GRK6 and β-arrestin-2 proteins in B cells from *Commd8*Δ, *Commd3*Δ (Δ), and littermate control (Ctrl; $Commd8^{f/f}Mb1^{+/+}$ or $Commd3^{f/f}Mb1^{+/+}$) mice. **(E)** IP assay for the interaction of Myc-tagged COMMD3 with HA-tagged GRK6 or GRK2 in 2PK-3 cells expressing Flag-tagged CXCR4 after stimulation with CXCL12. **(F)** IP assay for the interaction of Myc-tagged COMMD8 (left) or COMMD3 (right) with HA-tagged GRK5 was analyzed as in E. HA-tagged GRK6 served as a positive control. **(G)** IP assay for the interaction of Flag-tagged CXCR4 with HA-tagged GRK6 in vector-transfected control and COMMD3-deficient (sg*Commd3*) 2PK-3 cells stimulated with CXCL12. **(H)** IP assay for the interaction of Flag-tagged CXCR4 with Myc-tagged COMMD8 in vector-transfected control and GRK6-deficient (sg*Grk6*) 2PK-3 cells stimulated with CXCL12. Data are representative of two (D) or three (E and F) independent experiments. Error bars represent the mean ± SD of three (A–C and H) or four (G) independent experiments, and representative blots are shown. *, P < 0.05; **, P < 0.01. The P values were obtained by two-tailed unpaired *t* test. coIP, coimmunoprecipitation.
